# Equal abundance of summertime natural and wintertime anthropogenic Arctic organic aerosols

**DOI:** 10.1038/s41561-021-00891-1

**Published:** 2022-02-28

**Authors:** Vaios Moschos, Katja Dzepina, Deepika Bhattu, Houssni Lamkaddam, Roberto Casotto, Kaspar R. Daellenbach, Francesco Canonaco, Pragati Rai, Wenche Aas, Silvia Becagli, Giulia Calzolai, Konstantinos Eleftheriadis, Claire E. Moffett, Jürgen Schnelle-Kreis, Mirko Severi, Sangeeta Sharma, Henrik Skov, Mika Vestenius, Wendy Zhang, Hannele Hakola, Heidi Hellén, Lin Huang, Jean-Luc Jaffrezo, Andreas Massling, Jakob K. Nøjgaard, Tuukka Petäjä, Olga Popovicheva, Rebecca J. Sheesley, Rita Traversi, Karl Espen Yttri, Julia Schmale, André S. H. Prévôt, Urs Baltensperger, Imad El Haddad

**Affiliations:** 1 Laboratory of Atmospheric Chemistry, Paul Scherrer Institute, Villigen, Switzerland; 2 Multiphase Chemistry Department, Max Planck Institute for Chemistry, Mainz, Germany; 3 Center for Atmospheric Research, University of Nova Gorica, Ajdovščina, Slovenia; 4 Department of Civil and Infrastructure Engineering, Indian Institute of Technology Jodhpur, Jodhpur, India; 5 Datalystica Ltd, Villigen, Switzerland; 6 Norwegian Institute for Air Research (NILU), Kjeller, Norway; 7 Department of Chemistry ‘Ugo Schiff’, University of Florence, Florence, Italy; 8 Institute of Polar Sciences, ISP-CNR, Venice-Mestre, Italy; 9 National Institute for Nuclear Physics (INFN), Florence Division, Florence, Italy; 10 Environmental Radioactivity Laboratory, NCSR Demokritos, Athens, Greece; 11 Department of Environmental Science, Baylor University, Waco, TX USA; 12 Joint Mass Spectrometry Centre, Helmholtz Zentrum München, München, Germany; 13 Climate Research Division, Environment and Climate Change Canada, Toronto, Canada; 14 Department of Environmental Science, iClimate, Aarhus University, Roskilde, Denmark; 15 Atmospheric Composition Research, Finnish Meteorological Institute, Helsinki, Finland; 16 Institute of Environmental Geosciences, Université Grenoble Alpes, CNRS, Grenoble, France; 17 The National Research Centre for the Working Environment, Copenhagen, Denmark; 18 Institute for Atmospheric and Earth System Research/Physics, University of Helsinki, Helsinki, Finland; 19 Skobeltsyn Institute of Nuclear Physics, Lomonosov Moscow State University, Moscow, Russia; 20 Extreme Environments Research Laboratory, École Polytechnique Fédérale de Lausanne, Lausanne, Switzerland

**Keywords:** Atmospheric chemistry, Atmospheric chemistry, Atmospheric chemistry

## Abstract

Aerosols play an important yet uncertain role in modulating the radiation balance of the sensitive Arctic atmosphere. Organic aerosol is one of the most abundant, yet least understood, fractions of the Arctic aerosol mass. Here we use data from eight observatories that represent the entire Arctic to reveal the annual cycles in anthropogenic and biogenic sources of organic aerosol. We show that during winter, the organic aerosol in the Arctic is dominated by anthropogenic emissions, mainly from Eurasia, which consist of both direct combustion emissions and long-range transported, aged pollution. In summer, the decreasing anthropogenic pollution is replaced by natural emissions. These include marine secondary, biogenic secondary and primary biological emissions, which have the potential to be important to Arctic climate by modifying the cloud condensation nuclei properties and acting as ice-nucleating particles. Their source strength or atmospheric processing is sensitive to nutrient availability, solar radiation, temperature and snow cover. Our results provide a comprehensive understanding of the current pan-Arctic organic aerosol, which can be used to support modelling efforts that aim to quantify the climate impacts of emissions in this sensitive region.

## Abstract (web summary)

Organic aerosols in the Arctic are predominantly fuelled by anthropogenic sources in winter and natural sources in summer, according to observations from eight sites across the Arctic

## Main

Organic aerosols (OAs) contribute to the Arctic aerosol mass near the surface^1–4^ and affect the local climate through direct aerosol–radiation interactions and by altering the cloud properties^5,6^. OAs interact with other aerosol components^7–9^, for example, black carbon or elemental carbon (EC) and sulfate, and can augment or offset their radiative forcing^10^. The Arctic OA indirect effect is estimated to be of a similar magnitude as that of the sulfate indirect effect, and much larger than the OA direct effect^11^. The magnitude of these effects depends on the OA physicochemical properties, sources and formation processes^12,13^, which are not traceable by satellites^14^, and hence surface observations are indispensable^15^. However, OAs have received little attention in the Polar Regions^16–18^, mainly because of measurement challenges, and hence their complex composition and sources are poorly understood^19,20^. Nevertheless, an increasing abundance of natural OAs in a warming Arctic is expected^21^ as a result of northward-expanding vegetation^22^, intensifying boreal forest fires^23,24^, decreasing sea-ice extent^25^ and thawing permafrost^26,27^. Enhanced OA emissions are also expected from increasing local anthropogenic emissions, which include oil and gas exploration, and shipping activities^28,29^.

Attempts to model the Arctic OA concentrations have been limited, with typical under-predictions in winter and/or spring, when haze can be omnipresent and persistent^30–33^. The formation of secondary organic aerosols (SOAs) from anthropogenic or natural sources in different seasons is poorly represented^32,34^. Of 16 models deployed in a recent AeroCom evaluation of the simulated annual aerosol optical depth in Polar Regions^32^, only 6 considered biogenic precursors, which can contribute to particle growth and so the size range of cloud condensation nuclei. Only in one of the models was methanesulfonic acid (MSA) considered, which resulted in an outlier Arctic aerosol optical depth in terms of both seasonal variability and year-long magnitude^32^. A recent study showed that natural OAs other than MSA may account for about half of the summertime Arctic OAs^33^. Biological emissions, often not considered in Arctic models, may be a missing source of ice-nucleating particles^2,35^ that is possibly further enhanced with the thawing permafrost^36^. The contribution of anthropogenic emissions, for example, from gas flaring^37^, to primary OA and SOA precursors is expected to vary substantially in space and time across the Arctic region but, unlike black carbon^38^, has been largely overlooked. Long-term ambient measurements of the Arctic OA composition are critically needed to identify its main sources and therefore are a first step for subsequent implementation into models. However, available studies^3,39–41^ are single-site, short-term^3,39^ or campaign-based^39,40^, and too infrequent to reveal seasonal patterns in the main sources of Arctic OAs^42^. Hence, Arctic aerosol source apportionment studies typically do not consider OAs^43,44^, or are limited to a few primary markers (for example, levoglucosan from biomass burning or EC) and radiocarbon measurements^45,46^. Therefore, current efforts have so far been unable to provide an understanding of the sources and formation pathways of the pan-Arctic OAs in different seasons.

## Determination of OA sources across the Arctic

**Fig. 1:**
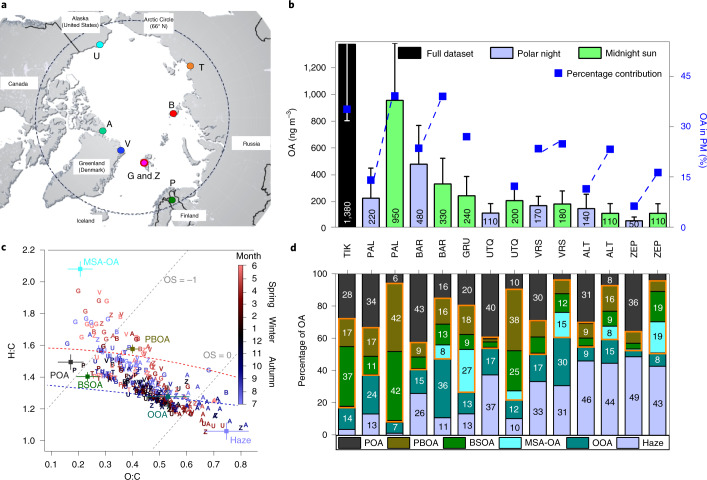
Sites, OA factors and chemical characteristics. **a**, Arctic political map showing the aerosol filter sampling stations (Supplementary Text 1 and Supplementary Table 1). A, Alert; B, Cape Baranova; G, Gruvebadet; P, Pallas-Matorova; T, Tiksi; U, Utqiaġvik; V, Villum Research Station; Z, Zeppelin. Adapted from Hugo Ahlenius/GRID-Arendal (https://www.grida.no/resources/8378). **b**, Station-specific average total OA mass concentrations (whiskers are 1 standard deviation, s.d., corresponding to sample-to-sample variability) based on a statistical analysis of the water-soluble fraction to obtain factor recoveries (Supplementary Text 4) in the polar night (winter) versus midnight sun (summer) periods (Supplementary Table 2), sorted in descending order of the station annual average, and percent contribution to the particulate mass (including non-sea salt sulfate, nitrate, ammonium, EC and estimated sea salt^63^). The dashed blue lines connect winter and summer contributions at each station (no winter samples for G and T, and U winter samples were not analysed for ions). **c**, Van Krevelen plot as a tool for compositional differentiation among samples: atomic O:C ratio versus H:C ratio of the Arctic AMS-PMF-based factors (Supplementary Fig. 2), and individual PMF input bulk samples (colour coded by month: 1, January, through to 12, December). Red and blue dashed curves refer to the triangle reported by Ng et al.^85^. Grey dashed lines denote two example oxidation states (OS). Error bars correspond to 1 s.d. from a bootstrap analysis (Methods and Supplementary Text 4). **d**, Spatial distribution and seasonal variability in the average factor percentage contributions to total OA (water-soluble (Supplementary Fig. 7); entire time-series (Supplementary Fig. 10)). Factors are sorted from bottom (Haze) to top (POA) based on their onset (see Fig. 3), starting from late winter for Haze. Primary OAs, POA + PBOA (top). Orange outline, sum of natural-dominated OAs.

Here we fill this critical knowledge gap by determining the spatial and seasonal distribution of individual OA classes (factors) across the Arctic (Fig. 1, Methods and Supplementary Text 1). This was achieved by offline aerosol mass spectrometer (AMS) measurements of nebulized aerosol water extracts^47^ from filter samples (Methods and Supplementary Text 2), followed by multisite positive matrix factorization (PMF) for OA source apportionment in relative terms, and subsequent quantification using externally measured water-soluble organic carbon (WSOC; see Methods). Factor identification is aided by organic marker, major ion and EC measurements (Methods and Supplementary Text 3). Source apportionment (Methods and Supplementary Text 4) results are also combined with concentration-weighted trajectory (CWT) analysis^48^ to determine the geographical origin of the OA factors (Methods and Supplementary Text 5). We performed the analysis of 350 (bi-)weekly composite samples from filters collected at eight observatories across the Arctic (Fig. 1a) at roughly overlapping periods in 2014–2019 (Supplementary Table 1), yielding unique results of annual cycles which included the periods of winter darkness and summer daylight (Supplementary Table 2). Using this methodology, we determined the anthropogenic and natural sources that drive the mass of primary OAs and SOAs in the pan-Arctic region in both winter and summer. Our work is the first systematic, concerted and multiseason, multistation effort with advanced analysis techniques, which goes beyond intensive observation periods and case-study work^20^.

**Fig. 2:**
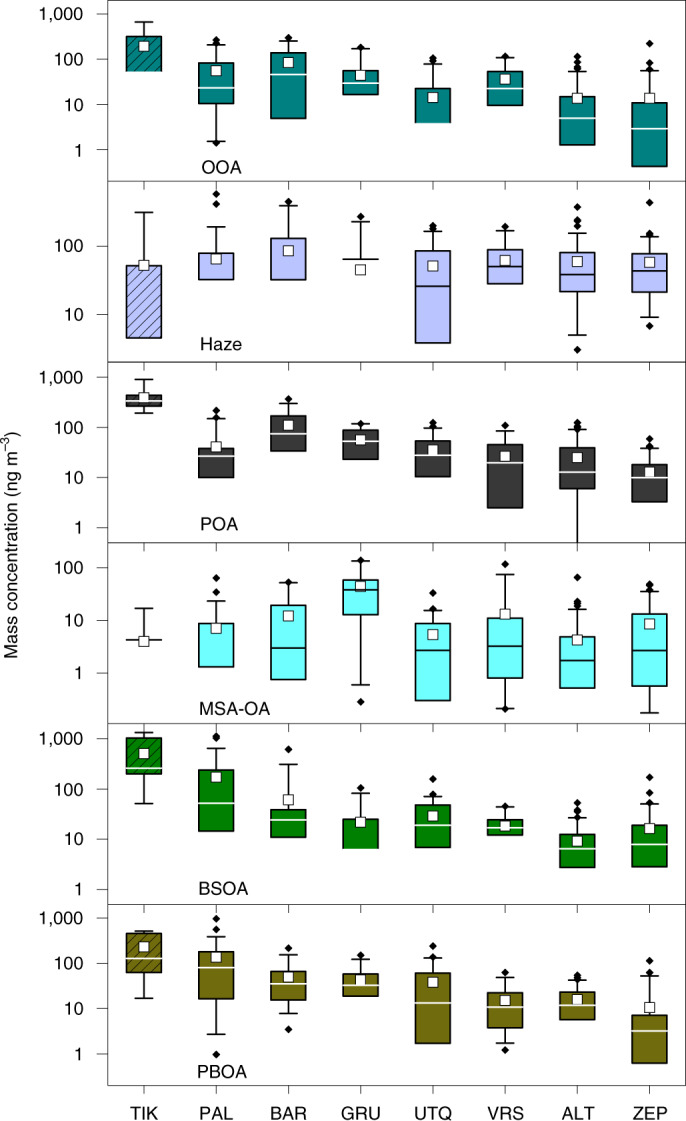
Station-specific yearly mass concentration of speciated Arctic OAs. Absolute OA factor concentrations at the different stations shown as box-and-whisker plots. The stations appear in the same order as in Fig. 1b, whereas the factors appear in the same order as discussed in the main text. Note the different range of *y*-axis values for the different factors. The lower whiskers are missing if associated values are out of scale. Boxes for TIK are hatched to indicate incomplete (inter)annual coverage. For WSOAs, see Supplementary Fig. 8. Horizontal line and box, yearly median and interquartile range; squares and whiskers, yearly mean and range within the 5th and 95th percentiles; diamonds, outliers.

**Fig. 3:**
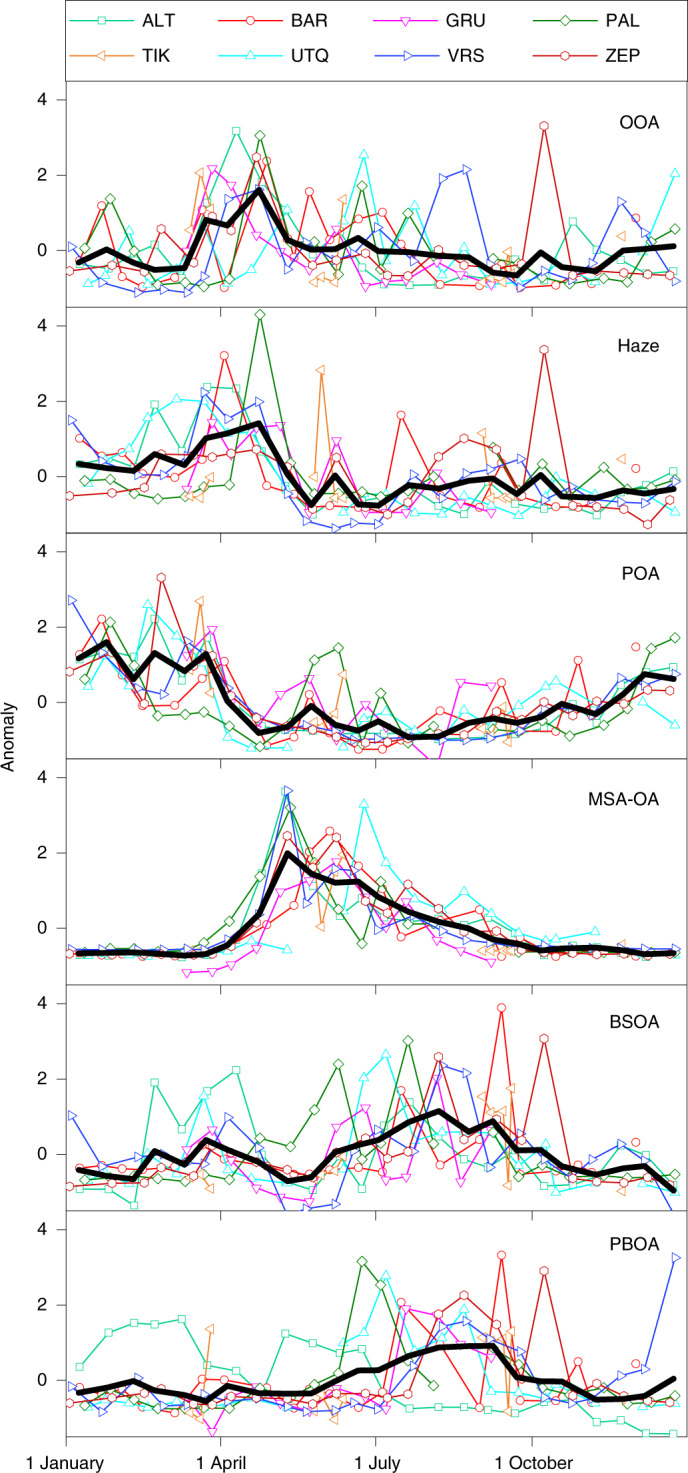
Seasonal variability of speciated pan-Arctic OAs. Standardized annual cycles for each OA factor at the different stations. Bi-weekly averaged data from multiple years for each station are merged into a single annual cycle (the sum of individual station values in each panel equals zero). The *y*-axis values (anomalies) were calculated using the absolute mass concentration values as: (value – station average)/s.d. of station. The thick black lines indicate the average annual cycle of each factor over all the stations (note that here the sum of the yearly values in each panel is not equal to zero).

**Fig. 4:**
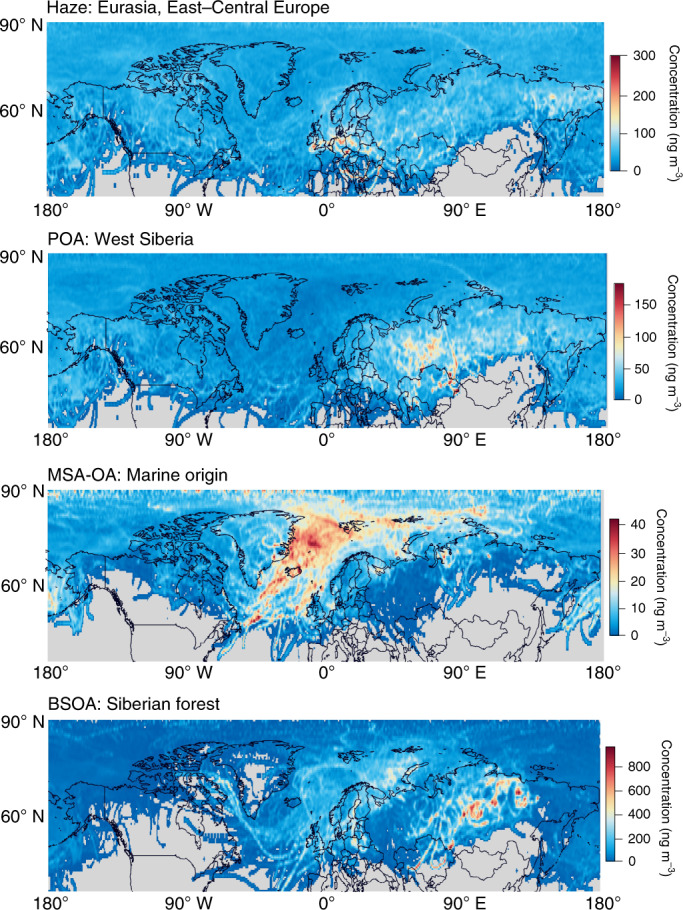
Major source regions of long-range transported Arctic OA factors. Merged results from the CWT-based back-trajectory (BT) analysis with ZeFir (Methods) at different Arctic stations (Supplementary Text 5 and Supplementary Fig. 11) showing long-term pan-Arctic hot spots of transported anthropogenic-dominated (Haze and POA) and natural-dominated (MSA-OA and BSOA) OA factors. The entire time series of each factor mass concentration at the different stations (time periods shown in Supplementary Table 1 and Supplementary Fig. 10) were used to create the maps (see Supplementary Text 5 for a discussion of the potential uncertainties in the source regions). The trajectories represent 5 days back in time for MSA-OA and (up to) 10 days for the other factors (Supplementary Text 5 and Supplementary Fig. 11). Colour scales indicate the water-soluble factor concentrations linked to the major source regions (‘long-range’ probability heat maps). The individual station results shown in Supplementary Fig. 11 were merged for each factor, except for POA, for which only six stations with winter data were considered here (no GRU and TIK), to indicate specific regions with intense gas-flaring activity during winter (for example, the Komi Republic, Khanty-Mansisk and Yamalo-Nenets autonomous districts in West Siberia). PBOA is expected to reside mainly in the coarse aerosol mode, and thus has a relatively short atmospheric lifetime (and hence more local and/or regional origins), and the formation of OOA might be linked to a prior accumulation of volatile organic compounds (thus probably not directly transported in the particle phase); therefore, the merged results for these factors are shown only in the Supplementary Information (Supplementary Fig. 11). The World Maps available with ZeFir are taken from Natural Earth Data.

The total OA was found to be a major aerosol fraction that typically contributes 10–40% to the total particulate mass at the different stations (Fig. 1b; the dataset range is 3–65%), with higher relative contributions in the summer. Although only water-soluble OA (WSOA) components were measured, the results represent the total OA by scaling the measurements by the water-soluble fraction of each factor (Methods and Supplementary Text 4). On average, WSOAs comprise 82 ± 4% of the total OA. Absolute OA concentrations were generally higher at the more continental stations, ~1.4 µg m^−3^ in Tiksi (TIK), ~0.4 µg m^−3^ in Cape Baranova (BAR) and 0.2 µg m^−3^ and 1.0 µg m^−3^ in Pallas-Matorova (PAL) in winter and summer, respectively, whereas typical concentrations at the other stations were lower (0.1–0.2 µg m^−3^). Figure 1c shows the O:C versus H:C ratios for all the samples measured by the AMS, which highlights the large variability in the OA chemical composition across stations and seasons. We determined six major OA factors (Methods and Supplementary Text 4, Supplementary Tables 3 and 4 and Supplementary Figs. 1–10). These include factors related to anthropogenic-dominated emissions, namely, oxygenated organic aerosol (OOA), Haze and primary-anthropogenic organic aerosol (POA), and to natural-dominated emissions, namely, MSA-related organic aerosol (MSA-OA), biogenic secondary organic aerosol (BSOA) and primary biological organic aerosol (PBOA). We could not relate any factor to fresh wildfire emissions in the spring or summer, probably because of multiple reasons such as the rapid aerosol ageing during transport^49^, emissions remaining aloft on ascent to the middle and/or upper troposphere (if they originate from North America and East and South Asia)^2,23,50^ and/or because this source becomes important only during short-term events. The atomic O:C versus H:C ratios of the six major OA factors (Fig. 1c) and their relative contributions across stations and seasons (Fig. 1d) are very distinct, and show the large diversity in the sources that drive the OA mass in the Arctic region. In the following, we discuss characteristic features of the factors, their spatial (Fig. 2) and seasonal (Fig. 3) variabilities, as well as their major source regions (Fig. 4).

## Anthropogenic-dominated OA factors

The OOA factor (O:C ~0.5) contains large oxygenated fragment ions (Supplementary Table 5). Its time-series shows a pan-Arctic enhancement during and after polar sunrise (Fig. 3), which potentially links this factor to oxidation products of volatile organic compounds that have accumulated during the polar night and form SOAs in the spring^25,51–53^. In this regard, a strong decrease in volatile organic compound concentrations was reported at PAL for April^54^. Although the OOA factor might be dominated by wintertime anthropogenic emissions, the contribution from natural sources during the summer cannot be excluded. The Haze factor contains more fragmented oxygenated ions (O:C ~0.75; Fig. 1c), builds up in late winter, peaks in spring (Fig. 3) and correlates with sulfate (Supplementary Table 6), similar to the profile and timing of the Arctic haze phenomenon^55^. Except for intermittent peaks at BAR, PAL and TIK, its yearly concentration is spatially homogeneous (Fig. 2), but with a certain temporally structured variability^56^ with the timing of the winter/spring peak occurring a few weeks later in time at most observatories as we move from west to east (Fig. 3), which indicates the effect of regional transport. This factor therefore becomes predominant in relative terms (Fig. 1d) at remote stations with lower OA loadings, that is, ~45% throughout the year at Alert (ALT) and Zeppelin (ZEP) (1st and 3rd quartiles, Q_1_–Q_3_ = 38–54%). Haze organics are associated with long-range atmospheric transport from Eurasia (Fig. 4). Therefore, the transport of pollutants from distant urbanized areas to remote environments contributes substantially to the formation of pan-Arctic SOAs (OOA and Haze; Q_1_–Q_3_ = 44–56% of the total OA during the polar night).

The POA factor is mainly composed of hydrocarbons (Supplementary Table 5) from primary anthropogenic emissions (O:C ~0.2), most probably related to gas flaring. POA has an H:C ratio of ~1.3 (Fig. 1c), lower than the H:C ratio (~1.8–1.9) of freshly emitted hydrocarbons detected at lower latitudes from traffic emissions^57,58^, which suggests a high contribution from unsaturated hydrocarbons. This factor is ubiquitous under dark and cold conditions, and peaks in December–March (Fig. 3) with a typical median pan-Arctic contribution to the total OA of 35% during the polar night (Fig. 1d, Q_1_–Q_3_ = 28–42%). In winter, POA correlates strongly with EC (Supplementary Fig. 12), which is reported to be of fossil origin^45,49^. The POA:EC ratio of ~1.1 is similar to the respective organic carbon (OC):EC ratios from oil and gas extraction emission estimates^59^. We found West Siberian locations (Fig. 4) as a major potential source region of the POA in winter (mainly at ALT, BAR, PAL and ZEP; Supplementary Fig. 11), similar to those previously found for surface black carbon^42,60,61^. The presence of POA at Gruvebadet (GRU) and TIK in the summer (Figs. 1d and Fig. 2) might indicate a more local origin at urban-type Arctic settlements.

## Natural-dominated OA factors

MSA-OA is characterized by the fragmentation pattern of MSA (for example, CH_3_ and CH_3_SO; Supplementary Table 5), which is produced from marine dimethylsulfide oxidation. It correlates strongly with MSA, which comprises on average 80% of the factor, as measured by ion chromatography (Supplementary Table 7). This factor consistently originates from marine regions (Fig. 4), and increases with solar radiation (Supplementary Fig. 14). The MSA-OA absolute concentrations (Fig. 2) and clear annual cycles (Fig. 3) are consistent among the stations, with much higher concentrations in May–June, the main season of phytoplankton blooms. The range of maximum concentrations at ALT and Utqiaġvik (UTQ) (~20–30 ng m^−3^) is comparable to those of previous measurements of MSA from these stations by ion chromatography^62,63^. The highest weekly averaged MSA-OA levels, which exceed 100 ng m^−3^, occur at GRU, Villum Research Station (VRS) and ZEP. Although at these stations MSA-OA is typically 22% (Q_1_–Q_3_ = 12–34 %) of the total OA during the midnight-sun period (Fig. 1d), this factor is neither the only nor the predominant natural OA component.

BSOA is linked to biogenic emissions, for example, mono- and/or sesquiterpenes and isoprene^64,65^, from forests, tundra, lakes, wetlands and marine waters. This factor dominates the variability of moderately oxygenated CHO fragments related to isoprene and terpene SOAs or, potentially, to biogenic stress responses (Supplementary Table 5), and correlates strongly (*R*^2^ = 0.81; Supplementary Fig. 13) with 3-methyl-1,2,3-butanetricarboxylic acid, a second-generation oxidation product of α-pinene. BSOA exhibits a clear annual cycle with enhanced values in June–September (Fig. 3), consistent with the exponential increase of biogenic precursor emissions with temperature^66^ (Supplementary Fig. 14). This factor dominates at PAL and TIK where it contributes, on average, 40% to the total OA in the summer (Fig. 1d, Q_1_–Q_3_ = 25–53%). At stations less affected by the boreal biome, BSOA appears in smaller but non-negligible amounts in the summer, and contributes 20%, on average, at UTQ and ZEP (Q_1_–Q_3_ = 11–28%) and ~9%, on average, at other stations. At PAL, we expect a more local and/or semiregional source^67,68^, whereas the episodic occurrence of BSOA in both Russian stations is linked to northward air mass transport from the Siberian forest (Fig. 4).

PBOA (O:C ~0.4, H:C ~1.6) is related to biological matter potentially from both terrestrial and marine origins, for example, fungal spores, bacteria, vegetative detritus, phytoplankton or fragments of it, and (ice) algae secretions^1,69,70^. It correlates with carbohydrate-related AMS fragments typically linked to primary biological compounds (Supplementary Table 5), and with arabitol and mannitol^71^ (*R*^2^ = 0.86; Supplementary Fig. 13), which are sugar-alcohols present in fungal spores and other biological matter. PBOA exhibits substantial concentration increases in July–September, to reach up to 1.0 µg m^−3^ at PAL (Fig. 2), and a distinct temporal evolution, peaking later than MSA-OA (Fig. 3). Similar to BSOA, PBOA exhibits higher relative contributions at lower latitude Arctic stations (~70° N), averaging one-third of the total OA at PAL and UTQ in the summer (Fig. 1d, Q_1_–Q_3_ = 26–47%). PBOA anticorrelates with the snow depth at PAL and VRS (where such data are available) and with the 30-year climate-normal average snowfall at UTQ (Supplementary Fig. 14). These findings indicate that maximum local biological activity is associated with the lack of snow covering the ground^72^, which causes entrainment of PBOA into the air.

## Implications of Arctic OA spatiotemporal variability

**Fig. 5:**
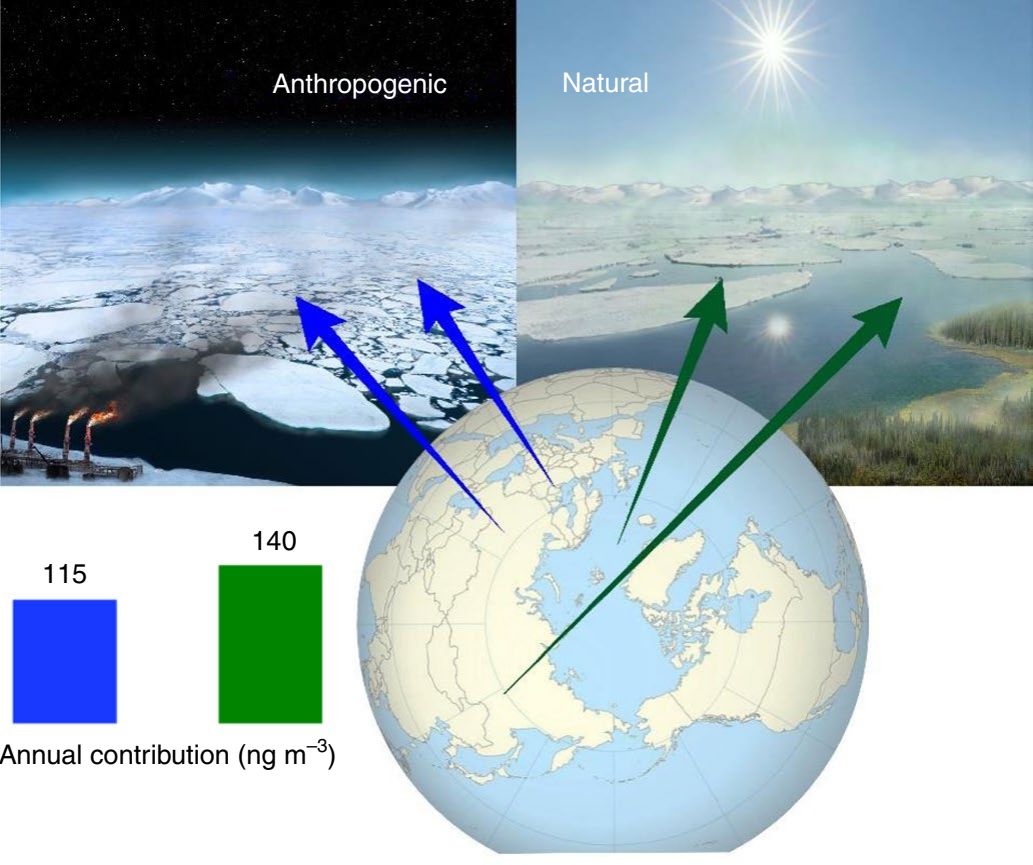
Conceptual overview of anthropogenic-dominated versus natural-dominated Arctic OAs. A conceptual image of anthropogenic-dominated and natural-dominated emissions that drive the OA mass in the Arctic in winter and summer, respectively. The most important geographical source regions are indicated by the arrows. Bars show the entire-dataset average contributions of nearly equally contributing summed anthropogenic-dominated (blue) and summed natural-dominated (green) organic components. Credits: Helen Cawley for the landscape drawing; map made using Natural Earth.

Overall, the pan‐Arctic OA composition and sources are not uniform. They are largely driven by the stations’ latitude (and altitude; Supplementary Fig. 15), favourable conditions for long-range atmospheric transport and distance to anthropogenic and natural aerosol sources, as well as the presence of light and snow cover. We show that secondary Arctic OAs, separated into various distinct subtypes, typically dominate the OA mass, although the contribution of primary OAs from both natural and anthropogenic emissions is often equally important (Fig. 1d). This finding is in line with lower-latitude OA source apportionment studies^12^. The current and future abundance of condensable organics could be critical for the cloud condensation nuclei budget and cloud properties. In the Arctic winter, the sea-ice extent is at its maximum, low-level clouds have a pronounced warming effect and surface pollutants can become trapped close to the ground due to temperature inversion^55^. In this period, the land-surface OA sources are predominantly anthropogenic and linked to both primary emissions and secondary processes. The widespread Haze organics are dominated by aged anthropogenic-dominated emissions transported mainly from Eurasia, peak during polar sunrise and abruptly decrease in May. Under cold and dark conditions, all the stations are affected by OA emissions related to oil or gas extraction activities, mainly in West Siberia. The Arctic amplification of temperature increase^73,74^ is more intense during winter^75^, and may be affected by aerosols altering the cloud properties. Hence, the future evolution of anthropogenic-dominated wintertime Arctic OA should be monitored from a climate perspective, especially in response to the development of effective emission control measures at lower latitudes^11,29^. In the summer, the decreasing anthropogenic pollution is replaced by natural OA emissions, with similar absolute concentrations. These emissions include marine SOAs from dimethylsulfide oxidation and little-explored primary biological emissions and biogenic SOAs. These collectively contribute to an increased importance of natural OA in the summertime aerosol particle mass in the inner Arctic^76^. We found an overall nearly equal yearly abundance (Fig. 5) of summed anthropogenic-dominated OAs (average of 115 ng m^−3^, Q_1_–Q_3_ = 20–100 ng m^−3^; 95th percentile, 435 ng m^−3^) and summed natural-dominated OAs (average of 140 ng m^−3^, Q_1_–Q_3_ = 40–160 ng m^−3^; 95th percentile, 445 ng m^−3^). This indicates that the typically lower total aerosol volume/mass in the summer versus winter or spring^77^ is due to species other than the total OA, which exhibits less of a seasonal cycle across the Arctic (Supplementary Fig. 16). The effects of anthropogenic and natural organics on cloud condensation nuclei and ice-nucleating particles, and potentially on the Arctic climate, have been largely unexplored. The importance of considering land coverage and biosphere–atmosphere exchanges^78,79^ for the response of natural Arctic OAs to warming is highlighted in Supplementary Fig. 14. A small increase in temperature results in a substantial (exponential) increase of BSOA, which identifies one of the potential feedback mechanisms in the Arctic. Also, extension of the ice-free season and expansion of snow-free areas^80^ may lead to enhanced airborne organics from biological activity and secondary marine emissions.

Currently, the Arctic system is in transition^21^, with long-range transported anthropogenic-dominated emissions (including sulfate) continuously decreasing due to better air quality regulations in the lower latitude regions. Meanwhile, natural emissions are expected to increase^81,82^, which probably enhances the magnitude and relative importance of the composition-dependent OA–cloud effect^11^. Our results provide the first understanding of the present-day year-long, pan-Arctic OA sources, which can be used for comparisons with past (for example, through ice-core archives^83^) and future measurements of these changing biogenic and anthropogenic emissions. Given practical difficulties in deploying multiple online AMS instruments for long time periods around the Arctic and the widespread availability of ambient filters, the measurement methodology and analysis techniques employed here are also applicable to emerging Arctic stations (for example, in far East Siberia^84^).

## Methods

### Filter sampling

Total suspended particulate matter or PM_10_ (particulate matter with aerodynamic diameter *d* < 10 µm) was collected on quartz fibre filters at eight stations around the Arctic (Fig. 1a). This study combined distinct locations affected by anthropogenic versus natural, marine versus terrestrial and local versus long-range transported emission sources at different seasons. PAL stands out from the other stations as it is in a sub-Arctic site influenced substantially by the boreal biome, which highlights differences with higher-latitude sites. Typically, at least one annual cycle is covered per station (Supplementary Table 1). The samples from GRU cover the spring and summer season (Supplementary Table 2) over two consecutive years, whereas the longest temporal coverage is almost four years (ALT). Although the data coverage at TIK is relatively less wide, this site experiences distinctly higher levels of pollution compared with that of other sites and includes the spring, summer and autumn periods over three consecutive years, and so covers a wide range of conditions. More details about the stations and filter sampling are provided in Supplementary Text 1, which includes a description of the conditions under which the filters were handled, transported between stations and/or labs and stored during and/or after sampling.

### AMS measurements and data analysis

The offline AMS technique was established by Daellenbach et al.^47^. Briefly, punches from the quartz fibre filter samples were extracted in ultrapure water (18.2 Mohm cm, with the total organic carbon (TOC) <3 ppb by weight). Teflon filters were tested but not measured (Supplementary Text 2). Typical quartz-fibre-filtered water-extracted organic concentrations were 2–3 µg C ml^–1^. The extracts were inserted into an ultrasonic bath for 20 min at 30 °C. Each sonicated sample was then filtered through a nylon membrane syringe (0.45 µm; Infochroma AG) and transferred to a ‘Greiner’ sample tube (50 ml). From the obtained solutions, aerosols were generated in synthetic air (80% volume N_2_, 20% volume O_2_; Carbagas) via an apex Q nebulizer (Elemental Scientific, Inc.) operated at 60 °C, dried by a Nafion dryer and directed into a long-time-of-flight AMS. Each sample was recorded for 480 s, with a collection time for each spectrum of ~40 s. Ultrapure water was measured for 720 s before and after each sample measurement. The technique was performed on ~370 extracts (samples and field blanks) in total.

For the data analysis, we used Squirrel v1.59B for the *m*/*z* calibration and baseline subtraction, and Pika v1.19B for high-resolution (HR) analysis, in the Igor Pro software package 6.37. The HR peak fitting was performed in the *m*/*z* range 12–191. After the peak fitting (fragments consisted of C, O, H, N and S), the isotope ions (and the inorganic fragments) were removed, and the average water-blank signal was subtracted from the average signal of the following sample. The resulting data matrix was the organic fraction mass spectra time series (normalized fragment ion intensity) and an error matrix that included the blank variability and measurement uncertainties. The signal-to-noise ratio (was >2.0 for 390 out of 578 fitted organic fragment ions with *m*/*z* up to 133, whereas it dropped to 1.9 on average for fragments with *m*/*z* between 134 and 180. The inorganic-salt artefact on the AMS CO_2_ (ref. ^86^) and CO fragment ion signal was also accounted for (Supplementary Text 2), but the resulting corrections were minor.

### Auxiliary measurements

Additional offline analyses were carried out (Supplementary Text 3); these included the measurement of EC and OC by a thermo-optical transmission method using a Sunset analyser, WSOC by a TOC analyser, major water-soluble ions (including MSA) by ion chromatography and organic markers (sugar-alcohols, sugars and organic acids) for selected samples. Several environmental parameters were retrieved as well, such as temperature, solar radiation and snow-depth data, which were averaged to match the time resolution of the filter sample composites measured by AMS (Supplementary Text 3).

### AMS-PMF analysis

#### Concept

The normalized organic mass spectra from the offline AMS measurements were analysed by PMF^87,88^ using the multilinear engine 2^89^. The aim was to derive source components (factors) that can be linearly combined to reproduce the observed time and chemical (mass spectral profiles) variations^90^ in the extracted organics. The separation of OA factors was therefore based on differences in their chemical composition and temporal behaviour, regardless of their original mixing state with other particulate matter components when suspended in the atmosphere. We did not use the absolute AMS signals for the quantification of the organic fraction, as they are affected by the AMS collection and transmission efficiencies and by the nebulization efficiency. Instead, we scaled the relative factor contributions determined by PMF using the TOC-based WSOC (see ‘Final AMS-PMF results’). Therefore, the total OA concentration is not affected by the collection efficiency. All extracted aerosol species are expected to be internally mixed in the nebulized particles, so the offline AMS collection efficiency should be the same for all identified OA factors^91,92^.

#### Methodology

The interface for the data processing was provided by the source finder toolkit^93^ (SoFi version 6.86) for Igor Pro (WaveMetrics, Inc.). PMF attempts to solve the bilinear matrix equation, *X*_*ij*_ = ∑*n* (*G*_*in*_*F*_*nj*_ + *E*_*ij*_), by following the weighted least-squares approach. In the case of AMS, *i* represents the time index, *j* is the fragment and *n* is the factor number. If *X*_*ij*_ is the matrix of the organic mass spectral data and *s*_*ij*_ the corresponding error matrix (including the blank variability and measurement uncertainties), *G*_*in*_ is the matrix of the factor time series, *F*_*nj*_ the matrix of the factor profiles and *E*_*ij*_ the model residual matrix, then PMF determines *G*_*in*_ and *F*_*nj*_ such that the ratio of the Frobenius norm of *E*_*ij*_ over *s*_*ij*_ is minimized. The allowed *G*_*in*_ and *F*_*nj*_ are always non-negative. The PMF input matrices (*X*_*ij*_ and *s*_*ij*_) here included data from all the stations (~350 samples) and 578 HR fragments with *m*/*z* up to 133 (or 1,029 HR fragments with *m*/*z* up to 191). All fragments with a signal-to-noise ratio below 0.2 were removed from the matrices, and those with a signal-to-noise ratio below 2.0 were down-weighted according to the recommendations of Paatero and Hopke (2003)^94^. For factor identification^90^, we used a combination of criteria where applicable. These include the factor seasonal cycle, fragmentation pattern, characteristic fragments, time series correlation with external markers, time series correlation with environmental parameters and the BT analysis.

#### Number of factors

Unconstrained PMF was performed for *n* = 3–15 factors to choose a ‘base case’ solution before the uncertainty analysis. Five random seed runs were performed for each *n* (65 runs in total). Preliminary diagnostics were then produced (Supplementary Fig. 1) to investigate the optimum *n* based on the explained variability of the input matrix (*Q*/*Q*_exp_ (exp, expected), scaled residuals) and the stability and/or interpretability of preliminary solutions among different runs for each *n*. All random seed runs provided essentially identical results (that is, the lowest *Q*/*Q*_exp_ relative s.d.) only for the 11-factor solution (the factor profiles are shown in Supplementary Fig. 2). The stability of this preliminary solution only (versus other *n*) among the different random seed runs is detailed in Supplementary Table 3. The *Q*/*Q*_exp_ of this average (‘base case’) 11-factor solution exhibited a random pattern in both dimensions (time series and variables) of the reconstructed PMF output matrix (350 samples and 578 HR fragments up to *m*/*z* 133). With regard to a partial exploration of the rotational ambiguity, the 11-factor solution was the most robust (Supplementary Text 4), as other factor solutions were less stable among the different random seed runs compared with that of *n* = 11 for certain factors (Supplementary Table 3).

#### PMF errors

The PMF model statistical and rotational uncertainty was assessed via a bootstrapping (BS) approach (Supplementary Text 4) applied to the entire dataset (by including PMF samples from the all stations and seasons). The BS approach generated new input matrices by randomly resampling samples from the original input matrix. Each newly generated PMF input matrix had a total number of samples equal to those of the original matrices, although some of the original filter samples were represented several times, and others were not represented at all. The resulting error interval represents temporal variations of the 11-factor profiles, random errors and errors in the modelling process, such as rotational ambiguity and a mis-specified number of factors^95^. Note that a preliminary BS analysis was performed also for the 10-factor solution, but the results were less stable than those of the 11-factor solution.

#### Retention of PMF factors related to ambient organic aerosols

Although the optimum PMF solution contains 11 factors, only the 6 factors presented in the main text and figures were interpreted to be related to the sampled OA. The remaining factors were associated with carbonate and (field) blanks (Supplementary Text 4). The carbonate-related (inorganic) factor may originate from sea spray and/or transported dust from lower latitudes or inner-Arctic sources. It exhibited an unusually high O:C of ~1.9 due to a very high $f_{{\rm{CO}}_2}$ of around 0.8 (Supplementary Fig. 2). We confirmed the presence of carbonate by HCl fumigation and offline AMS experiments for selected samples^96,97^. All the data presented are corrected for the carbonate (Supplementary Text 4). We separated a factor that was systematically associated with the filter substrate in terms of both the mass spectral fingerprint (factor profile) and the absolute mass concentrations at the different stations (Supplementary Text 4) and three other factors with contributions that could not be decoupled from the background signal of the filter (Supplementary Text 4 and Supplementary Fig. 4).

#### Final AMS-PMF results

The atomic ratios (O:C, H:C, N:C and S:C) and OM:OC ratios shown in Supplementary Fig. 2 were calculated for all the PMF input samples and for each AMS-PMF factor (for *m*/*z* up to 133), using the Analytical Procedure for Elemental Separation version 1.05 within Igor. The error bars in Fig. 1c correspond to 1 s.d. from the 100 BS runs. The median and interquartile range of the factor-specific (*k*) fractional contribution (Fc_*k*_) time series that resulted from the BS AMS-PMF analysis were converted into absolute WSOA mass concentrations by multiplying with the AMS-based (WS)OA:(WS)OC ratios of the different PMF-input samples (on carbonate subtraction) times the corresponding TOC-analyser-based WSOC values (total dissolved OC mass without any field blank subtraction): WSOA_*k*_ = Fc_*k*_ × WSOC × (WSOA/WSOC).

#### Recovery analysis performed using PMF

Recovery analysis was performed using PMF, following a simplified version of the approach of D. Bhattu et al. (personal communication; see Supplementary Text 4). The lowest water solubility was ~60–80% for POA, PBOA, Haze and OOA, whereas MSA-OA and BSOA can be considered fully water-soluble (Supplementary Text 4 and Supplementary Fig. 9). The AMS-PMF-based WSOA (*x*, the sum of six WSOA factors) versus the recovery-corrected total OA mass (*y*, the sum of six OA factors) time series were correlated with an *R*^2^ of 0.99 and a slope of 1.16, which indicated that the majority of OAs are water-soluble (the remaining factors were subtracted from both the *x* and *y* variables). The reconstructed AMS-PMF-based OC mass (*x*, the sum of all factors) versus the measured sunset OC (*y*) were correlated with an *R*^2^ of 0.87 and a slope of 1.14, which indicated a sufficient closure within the uncertainties. Although the elemental ratios shown in Supplementary Fig. 2 apply to water-extracted factors, the water solubility of Arctic OAs in this work was high (average ± 1 s.d. = 82 ± 4%), in agreement with previous estimates^98^, and does not vary significantly between locations and seasons. As a result, the data for the total OA shown in Figs. 1b,c and 2 are similar to those obtained for WSOA (Supplementary Figs. 7 and 8). The corresponding (median) total OA factor time series (Supplementary Fig. 10) were provided on conversion from WSOA values by applying median factor-specific recoveries, and were used as the result with regard to the factor time series. The median time series correlated highly with the base-case-solution time series (Supplementary Table 4).

#### Source-marker AMS fragments

We provide in Supplementary Table 5 specific fragments identified in our dataset as characteristic of specific sources, which were also identified in previous studies. These fragments were selected based on their highest contribution to these factors and the dominant contribution of these factors to these fragments.

#### Uncertainties

We briefly discuss here the PMF errors, the errors on the recovery, the errors on the relative ionization efficiency and the errors due to negative artefacts. The BS analysis indicated relative errors below 30% on average for median factor mass concentrations >50 ng m^−3^, with MSA-OA clearly being the least uncertain factor (Supplementary Text 4). The interquartile range of the median time series corresponds to errors from the BS runs without contingency for errors from the recovery analysis, by applying constant WSOA-to-OA factor-specific (median) conversion factors. We used the same relative ionization efficiency for all OA fractions. Given the recently reported relative ionization efficiencies for organic compounds of 1.2–1.8 (refs. ^99–101^), we expect the uncertainties that result from this simplification to be on the order of 20–30%, comparable to our modelling error estimates. Although the positive filter artefact is probably captured in this work through the identification of the field blank-related factor, the negative filter artefact (loss of semivolatile organics) could not be extensively assessed due to the current routine filter sampling strategies. In future analyses, it would be useful to address the negative filter artefact and other effects related to sample treatment, such as the effect of filter storage and revolatilization on water extraction, and to compare the offline results with concurrent online measurements.

### BT analysis for OA factor geographical origin assessment

BTs show the air mass history (origins and transport paths) and thus can provide information on the geographical location of potentially advected emissions at large geographical scales. Here, BT analysis (Supplementary Text 5) was performed to assess potential source locations of individual Arctic organic components or their precursors over the entire time period covered at each station. The trajectories were calculated backward for up to ten days using the HYSPLIT4 model with meteorological data from the Global Data Assimilation System with 1° resolution (GDAS1; ftp://arlftp.arlhq.noaa.gov/pub/archives/gdas1). The station coordinates correspond to the arriving location of each BT. We weighted the calculated BTs with the AMS-PMF-based factor time series using the CWT model to localize the air parcels responsible for high measured concentrations at the receptor site. For the CWT analysis, the Igor-based user interface ‘ZeFir’ was used^48^. Although the HYSPLIT trajectory module within ZeFir is currently limited to the use of regular GDAS meteorological field data files, it offers the option to run the model at any date and/or time (for example, an entire year). The average mass concentration of each factor in each sample was enlarged in ZeFir for 2, 7 or 14 days back in time (resolution of 6 h, except for the Russian stations at 4 h), depending on the average sampling time resolution at each station (for example, a 14 day enlargement for a bi-weekly sampling time resolution), and the corresponding enlarged BT results were averaged (within the software) to represent each individual sample. Air parcels rising at altitudes above 3 km a.g.l. were considered less likely to arrive at the receptor site and hence were discarded (a threshold altitude of 1.5 km did not produce substantially different results unless otherwise noted). We assessed the sensitivity of our results to the BT timescales showing that the resulting maps are robust and not too sensitive beyond certain lengths of time (Supplementary Text 5). Multisite merging is a powerful option in ZeFir to explore the bigger picture of source–receptor approaches, as combining several sites together leads to higher trajectory density values. Merged results (without normalization) are presented in Fig. 4 to indicate pan-Arctic hot spots with a greater accuracy than that of single-site results. No weighting function was applied to include the influence of less-frequent air mass trajectories from distant regions (‘long-range’ probability heat maps^102^). The main observations from individual station results are described in Supplementary Text 5.

## Online content

Any methods, additional references, Nature Research reporting summaries, source data, extended data, supplementary information, acknowledgements, peer review information; details of author contributions and competing interests; and statements of data and code availability are available at 10.1038/s41561-021-00891-1.

## Supplementary information

Supplementary Information Supplementary Text 1–5, Tables 1–7, Figs. 1–16 and references.

## Data Availability

The data used in this study^103^ is openly available at Zenodo (10.5281/zenodo.5775070). Please contact the corresponding authors when using the data.

## Code availability

Datalystica Ltd is the official distributor of SoFi Pro licenses. Origin was used for graph plotting. The open-source Igor-based package ZeFir is free to download at https://sites.google.com/site/zefirproject/download. The World Maps available with ZeFir are taken from Natural Earth Data.

## References

[1] Russell LM, Hawkins LN, Frossard AA, Quinn PK, Bates TS (2010). Carbohydrate-like composition of submicron atmospheric particles and their production from ocean bubble bursting. Proc. Natl Acad. Sci. USA.

[2] Willis MD, Leaitch WR, Abbatt JPD (2018). Processes controlling the composition and abundance of Arctic aerosol. Rev. Geophys..

[3] Nielsen IE (2019). Biogenic and anthropogenic sources of aerosols at the High Arctic site Villum Research Station. Atmos. Chem. Phys..

[4] Moschos, V. et al. Elucidating the present-day chemical composition, seasonality and source regions of climate-relevant aerosols across the Arctic land surface. *Environ. Res. Lett.*10.1088/1748-9326/ac444b (2022).

[5] IPCC *Climate Change 2013: The Physical Science Basis* (eds Stocker, T. F. et al.) (Cambridge Univ. Press, 2013).

[6] Bennartz R (2013). July 2012 Greenland melt extent enhanced by low-level liquid clouds. Nature.

[7] Kirpes RM (2018). Secondary sulfate is internally mixed with sea spray aerosol and organic aerosol in the winter Arctic. Atmos. Chem. Phys..

[8] Lohmann U (2020). Future warming exacerbated by aged-soot effect on cloud formation. Nat. Geosci..

[9] Moschos V (2021). Source-specific light absorption by carbonaceous components in the complex aerosol matrix from yearly filter-based measurements. Atmos. Chem. Phys..

[10] Yang Q, Bitz CM, Doherty SJ (2014). Offsetting effects of aerosols on Arctic and global climate in the late 20th century. Atmos. Chem. Phys..

[11] Sand M (2015). Response of Arctic temperature to changes in emissions of short-lived climate forcers. Nat. Clim. Change.

[12] Jimenez JL (2009). Evolution of organic aerosols in the atmosphere. Science.

[13] Moschos V (2018). Source apportionment of brown carbon absorption by coupling ultraviolet–visible spectroscopy with aerosol mass spectrometry. Environ. Sci. Technol. Lett..

[14] Tomasi C (2015). Aerosol remote sensing in polar regions. Earth Sci. Rev..

[15] Uttal T (2016). International Arctic systems for observing the atmosphere: an international polar year legacy consortium. Bull. Am. Meteorol. Soc..

[16] Quinn PK (2002). A 3-year record of simultaneously measured aerosol chemical and optical properties at Barrow, Alaska. J. Geophys. Res. Atmos..

[17] Hirdman D (2010). Long-term trends of black carbon and sulphate aerosol in the Arctic: changes in atmospheric transport and source region emissions. Atmos. Chem. Phys..

[18] Petäjä T (2020). Overview: integrative and comprehensive understanding on polar environments (iCUPE)—concept and initial results. Atmos. Chem. Phys..

[19] Tjernström M (2014). The Arctic Summer Cloud Ocean Study (ASCOS): overview and experimental design. Atmos. Chem. Phys..

[20] Abbatt JPD (2019). Overview paper: new insights into aerosol and climate in the Arctic. Atmos. Chem. Phys..

[21] Schmale J, Zieger P, Ekman AML (2021). Aerosols in current and future Arctic climate. Nat. Clim. Change.

[22] Pearson RG (2013). Shifts in Arctic vegetation and associated feedbacks under climate change. Nat. Clim. Change.

[23] Warneke C (2010). An important contribution to springtime Arctic aerosol from biomass burning in Russia. Geophys. Res. Lett..

[24] Brock CA (2011). Characteristics, sources, and transport of aerosols measured in spring 2008 during the Aerosol, Radiation, and Cloud Processes Affecting Arctic Climate (ARCPAC) Project. Atmos. Chem. Phys..

[25] Mungall EL (2017). Microlayer source of oxygenated volatile organic compounds in the summertime marine Arctic boundary layer. Proc. Natl Acad. Sci. USA.

[26] Kramshoj M (2018). Biogenic volatile release from permafrost thaw is determined by the soil microbial sink. Nat. Commun..

[27] Li H (2020). Overlooked organic vapor emissions from thawing Arctic permafrost. Environ. Res. Lett..

[28] Corbett JJ (2010). Arctic shipping emissions inventories and future scenarios. Atmos. Chem. Phys..

[29] Schmale J (2018). Local Arctic air pollution: a neglected but serious problem. Earth Future.

[30] Wang Q (2011). Sources of carbonaceous aerosols and deposited black carbon in the Arctic in winter–spring: implications for radiative forcing. Atmos. Chem. Phys..

[31] Browse J (2014). The complex response of Arctic aerosol to sea-ice retreat. Atmos. Chem. Phys..

[32] Sand M (2017). Aerosols at the poles: an AeroCom Phase II multi-model evaluation. Atmos. Chem. Phys..

[33] Croft B (2019). Arctic marine secondary organic aerosol contributes significantly to summertime particle size distributions in the Canadian Arctic Archipelago. Atmos. Chem. Phys..

[34] Mann GW (2014). Intercomparison and evaluation of global aerosol microphysical properties among AeroCom models of a range of complexity. Atmos. Chem. Phys..

[35] Wilson TW (2015). A marine biogenic source of atmospheric ice-nucleating particles. Nature.

[36] Creamean JM (2020). Thawing permafrost: an overlooked source of seeds for Arctic cloud formation. Environ. Res. Lett..

[37] Shah, T. Composition of organic gas emissions from flaring natural gas (Ramboll Environ, 2017); https://www.epa.gov/sites/production/files/2017-11/documents/organic_gas.pdf

[38] Eleftheriadis K, Vratolis S, Nyeki S (2009). Aerosol black carbon in the European Arctic: measurements at Zeppelin station, Ny-Ålesund, Svalbard from 1998–2007. Geophys. Res. Lett..

[39] Frossard AA (2011). Springtime Arctic haze contributions of submicron organic particles from European and Asian combustion sources. J. Geophys. Res. Atmos..

[40] Chang RYW (2011). Aerosol composition and sources in the central Arctic Ocean during ASCOS. Atmos. Chem. Phys..

[41] Leaitch WR (2018). Organic functional groups in the submicron aerosol at 82.5° N, 62.5° W from 2012 to 2014. Atmos. Chem. Phys..

[42] *AMAP Assessment 2015: Black Carbon and Ozone as Arctic Climate Forcers* (AMAP, 2015).

[43] Polissar AV, Hopke PK, Paatero P, Malm WC, Sisler JF (1998). Atmospheric aerosol over Alaska: 2. Elemental composition and sources. J. Geophys. Res. Atmos..

[44] Nguyen QT (2013). Source apportionment of particles at Station Nord, North East Greenland during 2008–2010 using COPREM and PMF analysis. Atmos. Chem. Phys..

[45] Winiger P (2019). Source apportionment of circum-Arctic atmospheric black carbon from isotopes and modeling. Sci. Adv..

[46] Rodríguez BT (2020). Seasonal cycle of isotope‐based source apportionment of elemental carbon in airborne particulate matter and snow at Alert, Canada. J. Geophys. Res. Atmos..

[47] Daellenbach KR (2016). Characterization and source apportionment of organic aerosol using offline aerosol mass spectrometry. Atmos. Meas. Tech..

[48] Petit JE, Favez O, Albinet A, Canonaco F (2017). A user-friendly tool for comprehensive evaluation of the geographical origins of atmospheric pollution: wind and trajectory analyses. Environ. Model. Softw..

[49] Barrett TE, Robinson EM, Usenko S, Sheesley RJ (2015). Source contributions to wintertime elemental and organic carbon in the Western Arctic based on radiocarbon and tracer apportionment. Environ. Sci. Technol..

[50] Stohl A (2006). Characteristics of atmospheric transport into the Arctic troposphere. J. Geophys. Res. Atmos..

[51] Kawamura K (1996). Source and reaction pathways of dicarboxylic acids, ketoacids and dicarbonyls in Arctic aerosols: one year of observations. Atmos. Environ..

[52] Asmi E (2016). Aerosol size distribution seasonal characteristics measured in Tiksi, Russian Arctic. Atmos. Chem. Phys..

[53] Kolesar KR (2017). Effect of Prudhoe Bay emissions on atmospheric aerosol growth events observed in Utqiaġvik (Barrow), Alaska. Atmos. Environ..

[54] Hakola H, Hellén H, Laurila T (2006). Ten years of light hydrocarbons (C_2_–C_6_) concentration measurements in background air in Finland. Atmos. Environ..

[55] Shaw G (1995). The Arctic haze phenomenon. Bull. Am. Meterol. Soc..

[56] Stone RS (2014). A characterization of Arctic aerosols on the basis of aerosol optical depth and black carbon measurements. Elementa Sci. Anthrop..

[57] Crippa M (2013). Identification of marine and continental aerosol sources in Paris using high resolution aerosol mass spectrometry. J. Geophys. Res. Atmos..

[58] Qin YM (2017). Impacts of traffic emissions on atmospheric particulate nitrate and organics at a downwind site on the periphery of Guangzhou, China. Atmos. Chem. Phys..

[59] Peters GP (2011). Future emissions from shipping and petroleum activities in the Arctic. Atmos. Chem. Phys..

[60] Popovicheva O (2019). East Siberian Arctic background and black carbon polluted aerosols at HMO Tiksi. Sci. Total Environ..

[61] Zhu C (2020). FLEXPART v10.1 simulation of source contributions to Arctic black carbon. Atmos. Chem. Phys..

[62] Sharma S (2019). A factor and trends analysis of multidecadal lower tropospheric observations of Arctic aerosol composition, black carbon, ozone, and mercury at Alert, Canada. J. Geophys. Res. Atmos..

[63] Moffett CE (2020). Long‐term trends for marine sulfur aerosol in the Alaskan Arctic and relationships with temperature. J. Geophys. Res. Atmos..

[64] Hu QH (2013). Secondary organic aerosols over oceans via oxidation of isoprene and monoterpenes from Arctic to Antarctic. Sci. Rep..

[65] Fu PQ (2013). Organic molecular composition of marine aerosols over the Arctic Ocean in summer: contributions of primary emission and secondary aerosol formation. Biogeosciences.

[66] Guenther A (2006). Estimates of global terrestrial isoprene emissions using MEGAN (Model of Emissions of Gases and Aerosols from Nature). Atmos. Chem. Phys..

[67] Ricard V (2002). Two years of continuous aerosol measurements in northern Finland. J. Geophys. Res. Atmos..

[68] Hellén H (2020). Sesquiterpenes dominate monoterpenes in northern wetland emissions. Atmos. Chem. Phys..

[69] Orellana MV (2011). Marine microgels as a source of cloud condensation nuclei in the high Arctic. Proc. Natl Acad. Sci. USA.

[70] Leck C, Bigg EK (2017). Biogenic particles in the surface microlayer and overlaying atmosphere in the central Arctic Ocean during summer. Tellus B.

[71] Bozzetti C (2016). Size-resolved identification, characterization, and quantification of primary biological organic aerosol at a European rural site. Environ. Sci. Technol..

[72] Bokhorst S (2016). Changing Arctic snow cover: a review of recent developments and assessment of future needs for observations, modelling, and impacts. Ambio.

[73] Najafi MR, Zwiers FW, Gillett NP (2015). Attribution of Arctic temperature change to greenhouse-gas and aerosol influences. Nat. Clim. Change.

[74] Acosta Navarro JC (2016). Amplification of Arctic warming by past air pollution reductions in Europe. Nat. Geosci..

[75] Box JE (2019). Key indicators of Arctic climate change: 1971–2017. Environ. Res. Lett..

[76] Becagli S (2016). Relationships linking primary production, sea ice melting, and biogenic aerosol in the Arctic. Atmos. Environ..

[77] Croft B (2016). Processes controlling the annual cycle of Arctic aerosol number and size distributions. Atmos. Chem. Phys..

[78] Arneth A (2010). Terrestrial biogeochemical feedbacks in the climate system. Nat. Geosci..

[79] Boy M (2019). Interactions between the atmosphere, cryosphere, and ecosystems at northern high latitudes. Atmos. Chem. Phys..

[80] Callaghan TV (2012). The changing face of Arctic snow cover: a synthesis of observed and projected changes. Ambio.

[81] Lindwall F, Svendsen SS, Nielsen CS, Michelsen A, Rinnan R (2016). Warming increases isoprene emissions from an arctic fen. Sci. Total Environ..

[82] Gali M, Devred E, Babin M, Levasseur M (2019). Decadal increase in Arctic dimethylsulfide emission. Proc. Natl Acad. Sci. USA.

[83] O’Dwyer J (2000). Methanesulfonic acid in a Svalbard ice core as an indicator of ocean climate. Geophys. Res. Lett..

[84] Petäjä T (2020). Research agenda for the Russian far East and utilization of multi-platform comprehensive environmental observations. Int. J. Digit. Earth.

[85] Ng NL (2011). Changes in organic aerosol composition with aging inferred from aerosol mass spectra. Atmos. Chem. Phys..

[86] Pieber SM (2016). Inorganic salt interference on CO_2_^+^ in Aerodyne AMS and ACSM organic aerosol composition studies. Environ. Sci. Technol..

[87] Paatero P, Tapper U (1994). Positive matrix factorization: a non-negative factor model with optimal utilization of error estimates of data values. Environmetrics.

[88] Ulbrich IM (2009). Interpretation of organic components from positive matrix factorization of aerosol mass spectrometric data. Atmos. Chem. Phys..

[89] Paatero P (1999). The multilinear engine—a table-driven, least squares program for solving multilinear problems, including the *n*-way parallel factor analysis model. J. Comput. Graph. Stat..

[90] Zhang Q (2011). Understanding atmospheric organic aerosols via factor analysis of aerosol mass spectrometry: a review. Anal. Bioanal. Chem..

[91] Bozzetti C (2017). Argon offline-AMS source apportionment of organic aerosol over yearly cycles for an urban, rural, and marine site in northern Europe. Atmos. Chem. Phys..

[92] O’Brien RE (2019). Ultrasonic nebulization for the elemental analysis of microgram-level samples with offline aerosol mass spectrometry. Atmos. Meas. Techn..

[93] Canonaco F, Crippa M, Slowik JG, Baltensperger U, Prévôt ASH (2013). SoFi, an IGOR-based interface for the efficient use of the generalized multilinear engine (ME-2) for the source apportionment: ME-2 application to aerosol mass spectrometer data. Atmos. Meas. Techn..

[94] Paatero P, Hopke PK (2003). Discarding or downweighting high-noise variables in factor analytic models. Anal. Chim. Acta.

[95] Reff A, Eberly SI, Bhave PV (2007). Receptor modeling of ambient particulate matter data using positive matrix factorization: review of existing methods. J. Air Waste Manag. Assoc..

[96] Karanasiou A (2011). On the quantification of atmospheric carbonate carbon by thermal/optical analysis protocols. Atmos. Meas. Techn..

[97] Vlachou A (2019). Development of a versatile source apportionment analysis based on positive matrix factorization: a case study of the seasonal variation of organic aerosol sources in Estonia. Atmos. Chem. Phys..

[98] Tomasi C (2007). Aerosols in polar regions: a historical overview based on optical depth and in situ observations. J. Geophys. Res. Atmos..

[99] Canagaratna MR (2015). Elemental ratio measurements of organic compounds using aerosol mass spectrometry: characterization, improved calibration, and implications. Atmos. Chem. Phys..

[100] Jimenez JL (2016). Comment on “The effects of molecular weight and thermal decomposition on the sensitivity of a thermal desorption aerosol mass spectrometer”. Aerosol Sci. Technol..

[101] Xu W (2018). Laboratory evaluation of species-dependent relative ionization efficiencies in the Aerodyne Aerosol Mass Spectrometer. Aerosol Sci. Technol..

[102] Potier E (2019). Characterizing the regional contribution to PM_10_ pollution over northern France using two complementary approaches: chemistry transport and trajectory-based receptor models. Atmos. Res..

[103] Moschos, V. Equal abundance of summertime natural and wintertime anthropogenic Arctic organic aerosols. *Zenodo*10.5281/zenodo.5775070 (2021). 10.1038/s41561-021-00891-1 PMC8916957 35341076

