# Supplementary material for: Equal abundance of summertime natural and wintertime anthropogenic Arctic organic aerosols
Source: Nat Geosci. 2022 Feb 28;15(3):196–202. doi: 10.1038/s41561-021-00891-1 (PMC8916957; doi:10.1038/s41561-021-00891-1)
Supplement: Supplementary file 1 — Supplementary Text 1–5, Tables 1–7, Figs. 1–16 and references. [file 41561_2021_891_MOESM1_ESM.pdf]

nature portfolio

https://doi.org/10.1038/s41561-021-00891-1

Supplementary information

Equal abundance of summertime natural and wintertime anthropogenic Arctic organic aerosols

In the format provided by the authors and unedited

Supplementary Information for "Equal abundance of summertime natural and wintertime anthropogenic Arctic organic aerosols" by Moschos et al.

Supplementary Text

Text S1. Description of filter sampling at the different Arctic stations

ALT: The site is officially called as "Dr. Neil Trivett Global Atmosphere Watch Observatory", which is an atmospheric baseline station operated by Environment & Climate Change Canada, located about 6 km SSW of Alert, Nunavut, on the northeastern tip of Ellesmere Island, about 800 km south of the geographic North Pole, locating on the shore of the Lincoln Sea, 15 km from the mouth of the Nares Strait. Alert Station (82°3'N, 62°2'W, 210 meters above sea level, m asl) is the most northerly site in the GAW Network. A custom-built high-volume aerosol sampler was used at the station to collect 164 samples between April 2015 and Dec. 2018, including field blanks. The sampler was installed at a walk-up deck, 4 m above the ground. Meteorological data (including air temperature and pressure) were collected on a 10 m tower connected to the laboratory. Flow rate was approximately 1.4 m^3 min^{-1} at STP condition. Quartz filters (Pall Life Sciences, PallFlex Membrane Filters, 8 x 10 in, USA) were pre-fired at 900 °C overnight and then shipped to Alert while already being loaded on cartridge in a clean room. During transport and storage, the filter-containing cartridges were wrapped by aluminum foil and they were inside of sealed plastic bags. Sampling time on those filters was either one week or two weeks, the latter being used from August to October (due to operational issues, no filter was sampled in July 2015). A total of 32 field blanks (roughly one for every four samples) were collected. After sampling, filters were stored (at room temperature ~20 °C) in their sampling cartridges (wrapped in aluminum foil inside sealed plastic bags) at the Alert station and shipped in cardboard boxes (containing 5 sampling cartridges each) to the Toronto lab at Environment Climate Change Canada where they were stored frozen at < -30°C. From these filters, a circular piece with 47 mm in diameter from the entire sampling area (17.8 cm x 22.8 cm) was cut in a clean room and put into a Petri Dish, which was wrapped in aluminum foil individually. The individual wrapped 164 filters were stored in several stainless containers with airtight lids and shipped to PSI for this study. Bi-weekly composites were prepared for offline AMS measurements, and several field blank filters from adjacent periods were combined to create seasonal composites from all years.

BAR: The research station Ice Base Cape Baranova was built in 2013. It is located near Cape Baranova on the coast of the Shokalsky Strait, which divides the Bolshevik and October Revolution islands of the archipelago Severnaya Zemlya (79°2'N 101°5'E; 30 m asl). The area adjacent to the station is characterized by the presence of sea ice, dome-shaped glaciers, and icebergs. An 18-month campaign was conducted from April 2015 to December 2016. PM_{10} was collected during the protocol times using the Comde Derenda sampling system with 2.3 m^3 h^{-1} pumped airflow. The sampling time was 48-72 h per sample depending on the season, atmospheric particulate loading, and weather conditions. Sampling volume was corrected to standard atmosphere conditions (0 °C, 1 atm). Teflon and quartz filters, heated prior to their use at 500 °C for 6 h, were used for elemental and ionic/carbon analyses. Upon removal from the sampling system, the filters were wrapped in aluminum foil, plastic tightly closed bag, and immediately put into a deep freeze. For transportation an additional plastic box was used with a tight lid. The duration of transportation was smaller than the duration of storage. More details are provided in Manousakas et al. (2020)[1].

GRU: Aerosol samples were collected by a low-volume TECORA ECHO-PM device operating at a flow of 38.33 L min^{-1} (2.3 m^3 h^{-1}) at STP condition, at the Gruvebadet Atmospheric Laboratory in Svalbard (78°9'N, 11°9'E; ~50 m asl), to collect 322 samples between February 2017 and December 2018. The sampler is located into Gruvebadet shelter, and the cut-off head is at about 4 m above the ground, easily accessible on the roof of the building. For both years, QFF from CHM chem lab group (Barcelona, Spain) were shipped to Ny Ålesund individually stored in polystyrene boxes. In 2017, 25 mm diameter QFF were used, while in 2018 47 mm diameter QFF were punched so to obtain 25 mm diameter membranes. Blank filters were periodically collected until the procedure was changed in 2018; by this change in sampling strategy, every sample can be related to its own blank[2]. Samplings were performed at daily resolution in 2017 and at 2-day resolution in 2018. After sampling, filters were stored in a freezer and then shipped in insulated boxes to the Department of Chemistry of the University of Florence, where they were kept frozen at -20 °C. From these filters, a punch of 1x1.5 cm size was cut from the whole circle sampling area (11.9 or 3.14 cm^2) in clean room and the remaining portion was put in a similar polyethylene box, sealed with plastic bags and shipped to PSI for this work. Composite samples of weekly typical resolution were prepared for AMS measurements.

PAL: The measurements were conducted between August 2018 and August 2019 at the sub-arctic Pallas site in Northern Finland (68°0'N, 24°2'E) which comprises of several stations. The aerosol samples were collected at the Pallas-Matorova station at an elevation of 340 m asl. The site is located approximately 170 km north of the Arctic Circle and the area is designated as a National Park. The vegetation type is coniferous forest. The long term monthly temperature average for January is -14 °C and for July 14 °C (Lohila et al., 2015) [3]. The site is considered as a background station. The site contributes to several international programs such as Arctic Monitoring and Assessment Programme (AMAP), European Monitoring and Evaluation Programme (EMEP), Global Mercury Observation System (GMOS), International Cooperative Programme on Integrated Monitoring of Air Pollution Effects on Ecosystems (ICP IM) and WMO Global Atmosphere Watch (GAW). The station is described in detail in Lohila et al. (2015) [3]. The aerosol samples were collected on the roof of Pallas-Matorova station with sampling height of approximately 4 m. Samples were collected to 47 mm QFF using MCZ µPNS1-LVS pump and a custom-built low-volume aerosol sampler with a PM_{10} head. The flow rate was approximately 38 L min^{-1}. An average of 55.2 m^3 of air was sampled per day. Collection time

was approximately one week but varied from six to nine days. Total sampled amount of air was typically between 330 and 500 m^3 per sample. After sampling, the filters were stored in a fridge at the station and shipped to PSI after one to three months in a Styrofoam box with a cooler.

TIK: Aerosol measurements at the International Hydro-Meteorological Observatory (HMO) Tiksi (71°6'N, 128°9'E) were taken at the Clean Air Facility (CAF), located 500 m from the Laptev sea coast and 5 km from the Tiksi settlement. The 20 m Tiksi meteorological flux tower is located around 300 m from the CAF. A total suspended particle (TSP) inlet was installed approximately 1.5 m above the CAF roof and 5 m above the ground. Aerosols were sampled at an air flow of ~45 L min^{-1} and during the protocol times. TSP was collected on 47 mm quartz fiber (Pallflex) and Teflon (Zefluor) filters for subsequent analyses in the laboratory. The low concentrations of ambient aerosols necessitated sampling times ranging from one day in November up to three days in September, to allow the loading to exceed the detection limit for relevant aerosol chemistry analyses. Sampling was performed in September and November of 2014, in March, May–June, and September 2015, and in June and September 2016. Upon removal from the sampling system, the samples were wrapped in aluminum foil, plastic tightly closed bag, and immediately put into a deep freeze. For transportation an additional plastic box was used with a tight lid. The duration of transportation was smaller than the duration of storage. More details are provided in Popovicheva et al. (2019).

UTQ: PM samples were collected on the North Slope of Alaska from June 2016 through September 2017, at the Climate Research Facility 7.4 km northeast of the village of Utqiaġvik (UTQ), Alaska (71°2'N, 156°4'W), 515 km north of the Arctic Circle. The site is approximately 1.6 km from the nearest coast. TSP samples were collected on QFF (Tissuquartz Filters 2500 QAT-UP; 20 x 25 cm) using Hi-Q high volume samplers ~10 m above ground level. The sampling duration was on average one week at a flow rate of 1.2 m^3 min^{-1}. Filters were removed from the sampler immediately after sample period had ended and were stored in a freezer on-site when not directly in use. QFFs were baked prior to sampling at 500 ^{o}C for 12 h and stored in aluminum foil packets and storage bags in a freezer before and after sampling. Field blanks were taken periodically throughout the sampling campaigns by placing an unsampled filter in a filter holder, placing it in the sampler momentarily, and then removing it and placing the filter in storage. Field blanks were treated in the same manner as sampled filters. Filters were shipped to and from the site in plastic bins in coolers cooled with blue ice packs.

VRS: Villum Research Station (VRS) is located in North Greenland (81°36'N, 16°40'W, 24 m asl). The atmospheric measurement site is located 2 km southeast of the Danish Military facility on a small peninsula (Princess Ingeborg peninsula). The region is characterized by a dry and cold climate with 188 mm of precipitation annually and an annual mean temperature of -16.9 °C. Dominant wind direction is from southwest and the observatory is most of the time upwind of the military outpost Station Nord. The annual average wind speed is 4 m s^{-1}. VRS is surrounded by sea ice with bare ground occasionally present in the summer and appearing more and more frequent the latest years. Polar sunrise is observed at the end of February, while polar night prevails from mid-October. A high-volume sampler (Digitel DHA-80) was operated in the air observatory at a flow rate of 500 L min^{-1} (STP) and regularly tested towards a transfer standard and adjusted. The inlet head was heated to avoid condensation. The HVS itself is placed indoor in a temperature-controlled room. The sample passes through a PM_{10} head located just on top of the HVS and sample air was collected on quartz fiber filters over one week corresponding to ~5 k m^3 of air. After sampling, the exposed filters are placed between two pieces of aluminum foil and placed in a rilsan bag and stored in the dark in a freezer at -20 ^{o}C. During transport by plane, the samples are still kept in the dark but the temperature is ambient (takes normally 2 days). After received at Aarhus University, the samples are stored again in a freezer at -20 ^{o}C.

ZEP: Sixty seven aerosol filter samples were collected at the Zeppelin Observatory (78°5'N, 11°5'E, 475 m asl) at Svalbard, Norway, between January 2017 and December 2018. The filter samples were collected using a Digitel high-volume sampler (PM_{10}) with a flow rate of 40 m^3 h^{-1} and a filter face velocity of 72.2 cm s^{-1}. The sampling inlet being situated 2.5 m above the roof level of the observatory and 7 m above the ground level. Aerosol particles were collected on pre-fired (850°C; 3 h) QFF (PALLFLEX Tissuequartz 2500QAT-UP; 150 mm in diameter) for 1 week, and according to the quartz fiber filter behind quartz fiber filter (QBQ) set up, thus providing dynamic field blanks. Back and front filters were mounted in pre-cleaned filter holders, wrapped in preheated aluminum foil, and locked in two Zip-lock polyethylene bags, taking place in NILUs clean room. Shipment from NILU to the Zeppelin Observatory and vice versa were made in aluminum boxes, typically ten filters in each parcel. During transport from NILU to the Zeppelin Observatory and back again, the parcel was kept in ambient air. At the Zeppelin Observatory, the filters were stored in a freezer (-18 ^{o}C) prior to and after being exposed. At NILU, exposed filter samples were stored in a freezer (-18 ^{o}C). Thermal-optical analysis (TOA) was performed using the Sunset Lab OC/EC Aerosol Analyzer, using transmission for charring correction and operated according to the EUSAAR-2 temperature program[4]. Aliquots were cut from each of the exposed filters in our clean room, wrapped in preheated aluminum foil, locked in two Zip-lock polyethylene bags, and shipped to PSI for analysis. Upon combination of certain front filters, 60 ZEP samples were measured with offline AMS at PSI.

Text S2. Additional information on the AMS measurements

We have tested Teflon filters vs. QFF, as Teflon filters were also available (alternating with QFF) from the Russian stations. However, the extraction efficiency of test Teflon filters in water was found to be significantly lower than that of the QFF, both filters being collected concurrently at PSI (to represent the same ambient sample, in order to assess only the potential effect of the substrate on extraction efficiency). In general, the filter substrate should be quartz fiber if the goal of the campaign includes OA monitoring, as the only offline method available to quantify the OC loadings involves the OA thermal decomposition, therefore a polymeric filter material could partially decompose leading to OC artifacts.

The inorganic-salt artifact on the AMS CO_2 and CO fragment ion signal[5] was also accounted for. We measured ammonium nitrate (AN) and ammonium sulfate (AS) standards (pure, without any filter extracts present) of three different concentrations (4, 12, 36 ppm) at the beginning and at the end of the campaign to correct data matrices for the inorganic AN/AS effects on both the CO and CO_2 signals, as a function of loading and time. The resulting "*b*" parameters (slopes) used to modify the fragmentation table were, b-AN: 0.012 (CO_2), -0.0032 (CO); b-AS: 0.0043 (CO_2), -0.0026 (CO), with no significant variability between the start and end of the measurement campaign (duration ~5 consecutive days). These "*b*" parameters were significantly lower than the average/median values by Pieber et al. (2016)[5], causing minor corrections.

Text S3. Auxiliary measurements
Additional offline analyses were carried out (using different punches/extracts than for the AMS measurements) to corroborate and validate the source apportionment results (Text S4), e.g. Fig. S12-S13 and Table S6. Elemental and organic carbon (Sunset-EC/OC) were quantified by thermal-optical analysis, following the EUSAAR2 protocol[4] (UTQ: NIOSH 5040; ALT: see next paragraph); water-soluble OC (WSOC) was measured by water extraction followed by catalytic oxidation and non-dispersive infrared detection of CO_2 using a total organic carbon (TOC) analyzer[6]. We measured major ions (including methanesulfonic acid, MSA) in selected samples by ion chromatography (IC)[7]. Organic markers were determined for selected samples: sugar-alcohols and sugars were measured by high-performance liquid chromatography (HPLC) associated with a fluorescence detector (LC 240 Perkin Elmer) and HPLC-pulsed amperometric detection[8]; organic acids were determined by LC-MS. IC-based MSA from the same filters (selected samples) was measured also by other laboratories. Correlation between IC-based MSA and TOC+AMS/PMF MSA-OA at different stations is provided in Table S7.

The EC and OC concentrations of the filters collected at ALT were analyzed by a thermal evolution protocol, developed at ECCC as EnCan-Total-900 (ECT9), to quantify the amount of OC and EC in carbonaceous aerosol and their $\delta^{13}C$ values[9,10]. The fractions were separated from each other, according to their degree of refractoriness. Specifically, carbon fractions were released by the ECT9 protocol in three steps: (1) OC at 550 °C for 600 seconds in pure He; (2) Pyrolyzed OC (PyOC) & carbonate carbon (CC) at 870 °C for 600 seconds in pure He; and (3) EC at 900 °C for 420 seconds in a mixture of 2 % O_2 with 98 % He. All fractions were fully oxidized to CO_2 by passing through a furnace containing MnO_2 maintained at 870 °C. For concentration determination, the CO_2 passed through a methanator at 500 °C, was converted to CH_4, and quantified with a flame ionization detector. Based on isotope measurements (^{14}C & ^{13}C), it was verified that the ECT9 protocol[11] effectively isolates OC or EC from complex mixtures of reference materials with an uncertainty of about 5 %.

Several environmental parameters were retrieved as well. Temperature data for TIK were obtained from Popovicheva et al. (2019)[12]. Temperature, solar radiation and snow depth data for VRS were obtained from the station website: https://villumresearchstation.dk/data/. Normal-climate average (1980-2010) snowfall data for UTQ were obtained from: http://akclimate.org/Climate/Normals; in June, July, and August the long-term absence of snow events at this site is evident, and PBOA concurrently increases significantly with decreasing snowfall, before becoming negligible starting from September. Electronic archive Arctic and Antarctic Research Institute (AARI) term meteorological and upper-air observations data at Research station "Ice Base Cape Baranova" 2013–2020 were obtained from: http://www.aari.ru/main.php?lg=1. Other data were retrieved from ebas (http://ebas.nilu.no) or measured within the current project. Data were averaged to match the time resolution of the filter sample composites measured by AMS.

Text S4. Additional information on the PMF analysis
<u>Number of factors</u>: Compared to n = 11, other factor solutions were less stable among the different random seed runs, for certain factors (Table S3): MSA-OA first appeared in the 8-factor solution but only in certain seed runs, while it was not identified in the 7-factor solution. Starting from n = 9, the time series of MSA-OA, BSOA, and PBOA became stable among the five random seed runs and were similar to those of the 11-factor solution in both absolute (slope close to 1.0) and relative terms (high R^2). This was also the case for POA and haze but starting from n = 10. OOA and CHN-rich also became stable starting from n = 10, but with different absolute contributions than for n = 11. The Field blank-related factor (see "Retention of PMF factors related to ambient organic aerosols" subsection below) remained mixed with other factors for $n \le 10$ and therefore was the last one separated in the 11-factor solution. For n > 11, the solutions resembled the 11-factor solution, except for specific factor splits, e.g. for n = 13, the CHN-rich factor was split into three factors, or the CHN-rich and BSOA factors were split into two factors each. Even though these splits might indicate some inherent variability in these components, the robust separation of associated distinct features was not possible by PMF and/or for this specific dataset and/or sample size. Therefore, with regard to a partial exploration of the rotational ambiguity, the analysis described above showed that the 11-factor solution was the most robust.

<u>Error/sensitivity analysis</u>: The uncertainty analysis approach followed in the present study is described as follows: a preliminary uncertainty analysis was carried out by performing 21 free BS runs, of which 16 were similar to the 11-factor "base case" solution. The rejected runs were related to (temporal) co-variability between certain factors (e.g. of POA and haze) and to one sample from TIK (24.09.2014) representing a pollution episode (highest organic mass among all stations) likely not explained by any factor. Unlike all other samples, the Q/Q_{exp} of this sample (only) remained high in the 11-factor solution. Therefore, this outlier sample was not included in any discussion/presentation. We have therefore

used from the 16 retained runs the 11-factor average mass spectral profiles' relative fragment ion intensities within one standard deviation (1 SD) to proceed with running BS 100 times for obtaining a modeling error estimate. By applying these constraints on the retrieved factor mass spectral profiles, we aimed at guiding the solution towards environmentally meaningful rotations[13], but without forcing the profiles into too narrow intervals that could potentially result in unrealistic/biased relative errors. We also introduced a "block BS" approach in these 100 runs, using a block length (l) of 7 consecutive samples, according to the semi-empirical criterion[14,15] $l = N^{1/3}$, where N was the sample size (~350). Therefore, 50 non-overlapping blocks were created (each block contained samples from one station). These were treated as single-sample blocks in the different resampling runs, which assisted in preserving the original time series structure to a certain extent, i.e. to account for the partial co-variability between e.g. haze and POA observed in the preliminary uncertainty analysis. Besides addressing co-variability issues, the blocked bootstrap strategy is also recommended when performing less than ~100 BS runs[16]. By following this approach, all 100 BS runs matched the base case solution with relative errors below 30 % on average for factor mass concentrations > 50 ng m^{-3}, with MSA-OA clearly being the least uncertain factor (Fig. S6). On the basis of comparable absolute mass concentrations, our relative errors were generally lower than those reported for > 100 ng m^{-3} in Daellenbach et al. (2017)[17].

In parallel, we assessed the sensitivity of the 11-factor solution by testing a complementary, independent approach conceptually similar to bootstrapping (not adopted as the main one eventually). This approach consisted of running PMF on randomly reduced datasets (< 350 samples in the input matrix) with variable sample size, i.e. 33 %, 50 %, 70 % and 85 % of the 350 samples, 5 times for each sample size (20 sensitivity runs in total), following the approach of Hedberg et al. (2005)[18]. The BSOA, MSA-OA and PBOA factors were identified (matching the base case) in all of these 20 sensitivity runs. At the same time, we observed similar features to those of the selected approach described before, i.e. one TIK sample affecting the output when randomly selected or not (e.g. misattribution of the CHN-rich, OOA and FB-related factors), and temporal co-variability, e.g. between the haze and POA factors, leading to imprecise factor identification. Overall 9 runs from this approach were matching the base case solution, with an increasing acceptance-to-rejection ratio by increasing the sample size (80 % solution acceptance for 85 % randomly reduced datasets). This is because reduced datasets may be explained by less than 11 factors (if constraints are not introduced) and indicates the importance of large PMF-input datasets in sufficiently capturing the variability in both the chemical composition and temporal trends. Without considering the high-loading TIK sample, 13/20 runs would instead be accepted. This exercise therefore provided further support of the observed stability of the solution, upon running BS with partial constraints on the retrieved mass spectral profiles, as eventually selected for this study and described above.

We also compared the 11-factor solution that included all *m/z* (up to 191) vs. the solution with *m/z* up to 133 (i.e. base case). The high correlation coefficients indicate excellent agreement for all OA factor time series (Table S4). No difference in the obtained PMF result was therefore observed by considering fragments with *m/z* > 133 or not. We note that the day-to-day relative contribution of these larger fragments to the total AMS signal by HR organics was 1.5 ± 0.4 %, although many of them exhibited high SNR (in the samples compared to the AMS water-blanks).

<u>Retention of PMF factors related to ambient organic aerosols</u>: We observed significant decreases in the f_{CO2} after fumigation for five selected samples with very high measured initial f_{CO2} (~0.75), which confirmed the presence of carbonate. By contrast, the decrease in f_{CO2} was not as significant for five other samples with lower carbonate-related factor content in relative terms (Fig. S3). We also found excellent agreement between the sample mass spectra after fumigation vs. mathematical subtraction of the carbonate-related factor as retrieved by PMF from the respective original measured AMS spectra (Fig. S3), which provides strong evidence of the sufficient removal of inorganic carbonate via fumigation. These support the mathematical subtraction approach used in the present study, especially considering that chemical damage of the organic content can occur upon fumigation[19]. The absolute concentrations of the remaining factors (median and IQR from the 100 BS runs) were corrected/rescaled to measured WSOC using a relative ionization efficiency of 1.4 for all organics[20] vs. 1.16 for carbonate[21], following the approach of Vlachou et al. (2019)[13].

We performed test AMS/PMF runs by including the field blank samples in the input matrix. The contributions of each organic factor in the blanks were then compared to the respective contributions in the samples (Fig. S4-S5). The absolute concentrations of the field-blank (FB)-related factor were not statistically different between the field blanks and the samples (Fig. S4). Further, the FB-related factor exhibits higher relative contributions at stations with non-pre-baked filters (one third -35 ± 17 %- vs. one fifth -21 ± 13 %- of the total signal, i.e. sum of the 11 factors, for pre-baked filters). Also, the FB-related factor profile from the base case solution correlated with one of the two factor profiles identified by applying PMF on the FB AMS spectra from various stations (scatter plot of 578 fragment relative intensities, R^2: 0.99; slope: 1.00; both profiles had identical f_{CO2} = 0.43). These provided both quantitative and qualitative support of a systematic association of the FB-related factor and its mass spectral fingerprint with the organic mass on the filter substrate at the different stations.

We identified two non-interpretable factors; one was S-rich (F1) and the other was N-rich (F2). Their combined profile fingerprint appeared to be separated as a second factor when applying PMF on the field blanks, with minor relative contributions compared to the FB-related factor but enhanced for ALT. Their combined contributions in the samples were overall significantly lower than those of the FB-related factor, amounting to less than 5 % of the total signal in the base case solution (sum of 11 factor absolute mass concentrations over all stations), with elevated contributions in

samples from ALT. In many samples these factors did not contribute with a real signal (Fig. S4), they exhibited relatively low absolute concentrations lacking temporal trend, and did not contain source-marker fragments nor correlated with available auxiliary data. Also, backward trajectory analysis did not provide any indication of specific source regions. These relatively minor factors were therefore not identified and thus were not considered for the discussion in the main text nor in the results presentation.

The CHN-rich factor (N:C ~0.11; Fig. S2) was rich in proteinaceous matter and dominated the variability of reduced N-containing fragments typically related to amino acids (Table S5). It exhibited yearly-average concentrations >100 ng m^{-3} in BAR, GRU and TIK. While the composition of this factor is well-understood, links to natural or anthropogenic primary emissions remain elusive. We hypothesize an association with combustion emissions (trash burning, landfills at TIK), biological matter (terrestrial dust, phytoplankton production, bacteria and biological degradation) and/or sea salt aerosol arising from the marine microlayer[22,23,24]. The latter can be supported by its fair correlation with estimated sea salt concentrations at GRU (R^2: 0.5), which could partially explain a lack of a clear temporal trend at the different stations. However, anthropogenic emissions cannot be excluded. This factor was relatively less defined and its contributions in the samples appeared to be distinguishable from the contributions in the blanks only for high mass concentrations (Fig. S4).

Final AMS/PMF result**:** Recovery analysis was performed using PMF, following a simplified version of the approach of D. Bhattu et al. (pers. Comm.). Briefly, the analysis was carried out on 265 samples where Sunset-OC data were available. Fifty BS runs were performed in total, where the output time series were constrained using all 11 factors (normalized median concentrations) within their IQR, as obtained from the 100 BS runs performed on the water-soluble fraction. In these 50 runs, the water-insoluble OC (Sunset-OC minus WSOC) time series was used as an additional variable in the PMF input matrix (scaled to WSOA of the input matrix). The recovery was then defined as the ratio of the output profiles' water-soluble-to-total signal ratio for each factor. The well-constrained resulting recoveries are shown in Fig. S9 for the six retained OA factors. The lowest water-solubility was ~60-80 % for POA, PBOA, haze and OOA, whereas MSA-OA and BSOA can be considered fully water-soluble. The median recoveries of the Carbonate-related, FB-related F1, F2 and CHN-rich factors were 90 %, 100 %, 99 %, 77 %, and 100 %, respectively.

Source-marker AMS fragments: We provide here specific fragments identified in our dataset as characteristic of specific sources, which were also identified in previous studies. These fragments were selected based on their highest contribution to these factors and the dominant contribution of these factors to these fragments. The numbers next to elements correspond to subscripts in standard chemical formulas, while the number behind each fragment indicates its *m/z* value. All AMS fragments correlating with each AMS/PMF factor full-dataset time series are listed in Table S5.

CHN-rich
Amino acid-related fragments[25,26]: CH4N (30), C2H4N (42), C2H3N (41, from proline upon COOH loss), C3H7 (43, from leucine), C2H6N (44, from alanine), C4H7 (55, from leucine), C3H6N (56 glutamic acid-specific), C3H8N (58), C4H8N (70, from proline upon COOH loss), C4H10N (72)
Alkyl fragments: C2H5 (29), C4H9 (57)

MSA-OA
CH & CS fragment ions indicating an MSA fragmentation pattern[27,28,29,30], with a characteristic high CH3 relative intensity: CH (13), CH2 (14), CH3 (15), CHS (45), CH2S (46), CH3S (47), CH2SO (62), CH3SO (63), CH2SO2 (78), CH3SO2 (79), CH4SO3 (96)

PBOA
Carbohydrate-related markers[25,31]: CHO (29), C3H3O (55, glycogen-specific), C3H4O (56, mannitol-specific), C3H9N (59), C2H4O2 (60), C2H5O2 (61), C3H5O2 (73), C5H5O (81), C4H5O2 (85, saccharose/glycogen-specific), C6H7O3 (127, glycogen-specific), C6H9O4 (glycogen-specific)
Heat stress-related markers[32,33]:
CH3O (31), CH4O (32), C2H2O2 (58), C2H3O2 (59), C3H6O2 (74)

BSOA
Biogenic stress response-related markers[32,33]:
C3H6O (58), C4H7O (71), C3H6O2 (74), C5H7O (83), C5H5O2 (97), C6H9O (97), C7H8O2 (124)
Isoprene SOA markers[34,35,36,37]: C3H3 (39), C4H5 (53)[39], C4H5O (69), C5H6O (82), C5H8O2 (100), C5H9O2 (101), C6H7O2 (111), C6H9O2 (113), C7H11O2 (127), C6H7O3 (127)
Terpene (a-pinene) SOA marker[38]: C8H11O4 (171)
MBTCA (2nd generation monoterpene SOA) signature[39]: C7H9O3 (141)
Other biogenic SOA-related fragments: C2H3O (43), C3H3O (55), C4H3O (67), C5H5O (81), C7H7O3 (139)

POA[26,40,41]

(Oxy-)PAH-related fragments: C6H2 (74), C6H3 (75), C6H4 (76), C7H3 (87), C7H4 (88), C7H5 (89), C8H5 (101), C9H6 (114), C10H6 (126), C10H7 (127), C10H8 (128), C11H7 (139), C12H6 (150), C12H7 (151), C12H8 (152), C13H9 (165), C12H8O (168), C14H8 (176), C13H8O (180)
Alkene/cycloalkane fragments: C3H5 (41), C4H7 (55), C5H9 (69), C6H11 (83), C7H13 (97)
Alkane fragments: C3H7 (43), C5H11 (71), C6H13 (85)
Other hydrocarbon fragments: C3H6 (42), C4H8 (56), C5H7 (67), C5H10 (70), C6H5 (77, aromatic), C6H12 (84), C7H7 (91, aromatic), C7H9 (93), C7H4O (104, phthalate), C9H13 (121), C8H5O2 (133, aromatic ion)

Text S5. Additional information on the back-trajectory analysis
The aim of this analysis was to identify long-term inner-Arctic vs. distant (transported) OA source components by coupling AMS/PMF with CWT. Based on the overall obtained results from all factors at all stations, and considering which factors are expected to be transported, we focused on the presentation and discussion of obtained results for haze, POA MSA-OA and BSOA, in order to further support their identification/interpretation, especially for haze. The atmospheric lifetime of pollutants is generally much longer in Arctic winter than at lower latitudes, due to slow dry/wet deposition. Tests were carried out with 10- vs. 14-d BTs for haze arriving at (remote) ALT (most likely component to be most aged over all sites), considering the large mean Arctic age of air in winter[42,43], but the result remained unchanged. Therefore, we did not extend the 10-d BTs in the subsequent main runs. We note that back-trajectories on the order of 10-d may emphasize the importance of Eurasia for Arctic pollution, while we cannot exclude transport over longer timescales and the importance of more southerly sources, e.g. in Asia. For MSA-OA prevalent in summer, 5-d BTs proved sufficient in identifying potential source origins in our study, in line with the relatively shorter mean Arctic age of air in summer[42]. We are aware of potential inaccuracies and artifacts related to, for instance, sparse Arctic weather data[44], complex orography around the station[45], surface effects[46] and generally shallow planetary boundary layer heights (low inversion layers) [47], however we have used long-term data and merged results (as detailed in the following) in an attempt to reduce their potential impact[48] on the main trends at a regional scale[49].

The main observations from individual station results shown in Fig. S11 were the following: i) haze (10-d BTs; 5-d for Sub-Arctic PAL) is largely transported from Europe and mainland Russia to the different Arctic stations; ii) POA (10-d BTs unless otherwise noted) has mainly Eurasian potential source regions, possibly except for GRU and TIK where a more local source influence can be expected (only summer samples were available), in which cases a trajectory-based approach conceptually fails to provide meaningful information; iii) MSA-OA (5-d BTs) exhibited a clear marine origin at all stations (negligible influence in TIK); iv) a similar (distant) source region in central Siberia is found for BSOA arriving at both BAR and TIK (Russian stations; 10-d BTs); a marine distant source at UTQ (10-d BTs) is not ruled out; more local/regional influence is expected at PAL (5-d BTs), in which case a trajectory-based approach conceptually fails to provide meaningful information. BT results for the BSOA factor in other stations were not considered/interpreted due to lack of response to temperature (see Fig. S14).

Supplementary Tables

Table S1. Filter sampling coverage.

Year: 2014 (Sep–Dec), 2015, 2016, 2017, 2018, 2019 (Jan–Aug) — Station	(bi-)weekly composites
Utqiagvik/Barrow	45
Alert	88
Villum	38
Zeppelin	60
Gruvebadet	33
Pallas	42
Cape Baranova	29
Tiksi	14
field blanks	23
**Sample Size (offline AMS)**	**372**

Table S2. Polar night (wintertime absolute darkness) and midnight sun (summertime continuous sunlight) periods at the different Arctic stations. Note that these time periods vary slightly from one station to another due to their different latitude (e.g. earlier onset and larger duration of wintertime darkness at higher-latitude stations).

**Station**	**Polar night**	**Midnight sun**
Alert	Oct. 14 – Feb. 28	Apr. 07 – Sep. 04
Baranova	Oct. 22 – Feb. 22	Apr. 22 – Aug. 22
Pallas	Dec. 10 – Jan. 22	Apr. 27 – Jul. 17
Tiksi	Nov. 19 – Jan. 24	May 11 – Aug. 03
Utqiaġvik	Nov. 19 – Jan. 23	May 11 – Aug. 01
Villum	Oct. 16 – Feb. 25	Apr. 09 – Sep. 02
Zeppelin/Gruvebadet	Oct. 26 – Feb. 15	Apr. 20 – Aug. 20

Table S3. Time series correlations between identified factors in various AMS/PMF solutions for different number of factors (from 7 to 13), and the respective factor time series from the base case 11-factor solution. Factors colored in grey (Carbonate, FB-related, F1, F2, CHN-rich) were interpreted to not be related to sampled organics or major OA sources.

	**11-factor** Runs (y) vs. 5-run avg. ("base case"; x)									
	Run #1		Run #2		Run #3		Run #4		Run #5	
	R^2	slope	R^2	slope	R^2	slope	R^2	slope	R^2	slope
Carbonate	0.99	1.01	0.99	0.98	0.99	0.99	0.99	1.03	0.99	1.03
FB-related	0.99	0.99	0.99	1.00	0.99	1.00	0.99	1.00	0.99	1.00
F1	1.00	1.02	1.00	0.96	1.00	0.99	1.00	1.01	1.00	1.01
F2	1.00	0.99	1.00	1.01	0.99	1.02	0.99	1.01	0.99	1.01
CHN-rich	1.00	1.00	1.00	0.99	0.99	0.99	1.00	0.99	1.00	0.99
BSOA	0.99	0.99	1.00	1.00	0.99	1.01	0.99	0.99	0.99	0.99
POA	0.99	1.02	0.99	0.98	0.99	0.99	0.99	1.01	0.99	1.01
OOA	1.00	0.99	1.00	0.99	0.99	0.97	1.00	0.99	1.00	0.99
PBOA	0.99	0.99	0.99	1.01	0.99	1.01	0.99	0.99	0.99	0.99
MSA-OA	1.00	0.99	1.00	1.01	1.00	1.03	1.00	0.97	1.00	0.97
Haze	0.99	1.03	0.99	1.02	0.99	1.11	0.99	1.07	0.99	1.06

	**10-factor** Runs (y) vs. 11-factor –base case (x)									
	Run #1		Run #2		Run #3		Run #4		Run #5	
	R^2	slope	R^2	slope	R^2	slope	R^2	slope	R^2	slope
Carbonate	0.86	0.95	0.86	0.95	0.88	0.97	0.87	0.98	0.87	0.98
FB-related	-	-	-	-	-	-	-	-		
F1	0.99	1.70	0.99	1.67	0.99	1.67	0.99	1.69	0.99	1.71
F2	0.99	1.07	0.99	1.06	0.99	1.06	0.99	1.07	0.99	1.06
CHN-rich	0.82	2.12	0.81	2.14	0.81	2.14	0.77	2.27	0.75	2.27
BSOA	0.91	1.16	0.91	1.15	0.91	1.22	0.92	1.09	0.91	1.08
POA	0.98	1.13	0.98	1.12	0.98	1.12	0.87	1.03	0.82	0.99
OOA	0.95	0.45	0.95	0.45	0.95	0.45	0.93	0.48	0.92	0.49
PBOA	0.99	0.99	0.99	0.99	0.99	0.99	0.99	1.00	0.99	1.01
MSA-OA	0.99	0.98	0.99	0.98	0.99	0.97	0.99	0.94	0.99	0.93
Haze	0.93	1.07	0.92	1.04	0.92	1.05	0.77	1.02	0.72	1.04

	**9-factor** Runs (y) vs. 11-factor –base case (x)									
	Run #1		Run #2		Run #3		Run #4		Run #5	
	R^2	slope	R^2	slope	R^2	slope	R^2	slope	R^2	slope
Carbonate	0.70	0.95	0.67	0.93	0.70	0.96	1.02	0.81	0.81	0.99
FB-related	-	-	-	-	-	-	-	-	-	-
F1	0.99	1.53	0.99	1.52	0.99	1.58	0.99	1.35	0.99	1.02
F2	0.97	0.98	0.97	0.98	0.98	0.97	0.98	1.04	0.97	1.04
CHN-rich	0.73	1.26	0.75	1.33	0.77	1.36	0.93	2.05	0.95	1.93
BSOA	0.88	1.33	0.88	1.24	0.86	1.34	0.93	1.15	0.94	1.16
POA	0.71	1.45	0.72	1.46	0.72	1.44	0.79	1.50	0.76	1.49
OOA	-	-	-	-	-	-	0.70	0.84	0.60	0.78
PBOA	0.80	1.21	0.82	1.14	0.84	1.16	0.89	0.99	0.88	1.01
MSA-OA	0.99	1.25	0.99	1.25	0.99	1.24	0.99	1.24	0.99	1.25
Haze	0.92	1.27	0.91	1.28	0.92	1.23	-	-	-	-

	**8-factor** Runs (y) vs. 11-factor –base case (x)									
	Run #1		Run #2		Run #3		Run #4		Run #5	
	R^2	slope	R^2	slope	R^2	slope	R^2	slope	R^2	slope
Carbonate	0.76	1.01	0.76	1.01	0.76	1.01	0.84	0.97	0.71	0.94
FB-related	-	-	-	-					-	-
F1	0.99	1.27	0.99	1.27	0.99	1.26	0.94	1.30	0.94	1.50
F2	0.77	1.51	0.78	1.51	0.77	1.51	0.83	1.26	0.87	1.17
CHN-rich	0.88	1.84	0.88	1.85	0.88	1.84	0.93	1.80	0.73	1.27
BSOA	0.67	1.04	0.67	1.03	0.68	1.04	0.90	1.25	0.89	1.28
POA	-	-	-	-	-	-	0.66	1.33	0.64	1.41
OOA	0.76	1.42	0.75	1.42	0.76	1.42	0.57	0.67	-	-
PBOA	0.85	0.99	0.86	0.94	0.86	0.99	0.38	0.88	0.51	1.17
MSA-OA	0.99	1.23	0.99	1.28	0.99	1.20	-	-	-	-
Haze	-	-	-	-	-	-	-	-	0.83	1.23

	**7-factor** Runs (y) vs. 11-factor –base case (x)									
	Run #1		Run #2		Run #3		Run #4		Run #5	
	R^2	slope	R^2	slope	R^2	slope	R^2	slope	R^2	slope
Carbonate	0.73	0.95	0.71	0.87	0.78	0.99	0.72	0.90	0.71	0.87
FB-related	-	-	-	-	-	-	-	-	-	-
F1	0.94	1.51	0.94	1.53	0.94	1.60	0.94	1.46	0.94	1.50
F2	0.68	2.09	0.71	2.46	0.67	1.53	0.72	2.36	0.72	2.43
CHN-rich	0.87	1.79	0.86	1.39	0.87	1.79	0.89	1.48	0.87	1.43
BSOA	0.72	1.11	0.70	1.29	0.73	1.14	0.63	1.25	0.69	1.27
POA	-	-	0.56	1.37	-	-	0.56	1.21	0.57	1.34
OOA	0.84	1.35	-	-	0.84	1.34	-	-	-	-
PBOA	0.35	0.85	0.36	0.76	0.36	0.85	0.42	0.84	0.34	0.75
MSA-OA	-	-	-	-	-	-	-	-	-	-
Haze	-	-	-	-	-	-	-	-	-	-

	**12-factor** Runs (y) vs. 11-factor –base case (x)									
	Run #1		Run #2		Run #3		Run #4		Run #5	
	R^2	slope	R^2	slope	R^2	slope	R^2	slope	R^2	slope
Carbonate	0.97	0.95	0.99	0.98	0.97	0.98	0.96	0.94	0.99	0.97
FB-related	0.97	0.98	0.99	1.03	0.97	0.94	0.94	0.94	0.94	1.03
F1	0.99	1.24	0.99	0.99	0.99	1.22	0.99	1.33	0.99	0.88
F2	0.97	0.82	0.99	0.99	0.98	0.84	0.96	0.79	0.99	0.97
CHN-rich	0.91	0.87	0.57	0.63	0.77	0.81	0.95	0.95	0.47	0.72
BSOA	0.91	0.85	0.99	0.98	0.99	0.99	0.74	0.77	0.99	0.98
POA	0.83	0.95	0.95	0.88	0.90	0.89	0.88	0.99	0.94	0.87
OOA	0.98	0.80	0.97	0.79	0.99	0.99	0.98	0.78	0.96	0.77
PBOA	0.92	0.78	0.99	0.93	0.96	0.82	0.92	0.77	0.99	0.93
MSA-OA	0.99	0.99	0.99	0.92	0.99	0.99	0.99	0.96	0.99	0.97
Haze	0.90	0.96	0.99	1.07	0.98	1.11	0.79	0.82	0.99	0.93

	**13-factor** Runs (y) vs 11-factor –base case (x)									
	Run #1		Run #2		Run #3		Run #4		Run #5	
	R^2	slope	R^2	slope	R^2	slope	R^2	slope	R^2	slope
Carbonate	0.99	0.96	0.94	0.95	0.95	0.96	0.90	0.88	0.94	0.94
FB-related	0.95	0.94	0.89	0.90	0.90	0.87	0.95	0.95	0.90	0.88
F1	0.99	0.99	0.99	1.53	0.99	0.88	0.99	0.88	0.99	0.88
F2	0.97	0.83	0.95	0.75	0.97	0.87	0.97	0.87	0.97	0.87
CHN-rich	-	-	-	-	-	-	-	-	-	-
BSOA	0.95	0.96	0.82	0.67	0.84	0.88	0.83	0.87	0.83	0.87
POA	0.87	0.80	0.87	0.97	0.91	0.84	0.91	0.85	0.90	0.86
OOA	0.84	0.62	0.97	0.72	0.81	0.82	0.81	0.82	0.81	0.82
PBOA	0.96	0.87	0.97	0.79	0.95	0.92	0.95	0.92	0.95	0.92
MSA-OA	0.99	0.92	0.99	0.87	0.99	0.90	0.99	0.90	0.99	0.90
Haze	0.98	1.11	0.73	0.64	0.97	1.21	0.97	1.21	0.96	1.22

Table S4. Same Table S3, but for the 11-factor solution by including fragments up to *m/z* 133 vs. all *m/z*. The excellent agreement between the 11-factor base case solution and the median factor time series based on the 100 BS runs is also demonstrated.

	***n* = 11;** all *m/z* (y) vs. *m/z*<133, base case (x)	
	R^2	slope
Carbonate-related	0.96	1.01
FB-related	0.94	0.94
F1	0.99	1.05
F2	0.99	1.07
CHN-rich	0.99	1.14
BSOA	0.97	0.89
POA	0.99	1.05
OOA	0.99	1.01
PBOA	0.99	0.91
MSA-OA	0.99	1.09
Haze	0.96	1.01

	***n* = 11;** 100 BS-median (y) vs. base case (x)	
	R^2	slope
Carbonate-related	0.97	1.03
FB-related	0.96	0.96
F1	0.99	1.46
F2	0.99	1.10
CHN-rich	0.99	1.11
BSOA	0.99	1.00
POA	0.98	1.06
OOA	0.98	0.84
PBOA	0.99	1.05
MSA-OA	0.99	1.02
Haze	0.97	1.07

Table S5. Correlation of 266 AMS fragment ion time series (used in the PMF input) with the corresponding full-dataset time series of seven PMF-output OA factors (identified "marker" fragments are indicated with **bold**). Fragments were classified according to the Pearson's *r* correlation coefficients, and then they were ordered by increasing *m/z* (25 HR fragments with SNR < 2.0 are indicated with *italics*). Fragments correlating with two factors are indicated with the color of the other factor.

**MSA-OA**					
0.50 ≤ r < 0.70		0.70 ≤ r < 0.90		r ≥ 0.90	
m/z	*fragment ion*	*m/z*	*fragment ion*	*m/z*	*fragment ion*
46.9955	**CH3S**	13.0078	**CH**	15.0235	**CH3**
75.9441	*CS2*	14.0156	**CH2**	44.9799	**CHS, CH2S**
95.9881	**CH4SO3**	77.9775	**CH2SO2**	61.9826	**CH2SO**
		78.9854	**CH3SO2**	62.9905	**CH3SO**

**PBOA**					
0.50 ≤ r < 0.70				0.70 ≤ r < 0.90	
m/z	*fragment ion*	*m/z*	*fragment ion*	*m/z*	*fragment ion*
26.0156	C2H2	80.0262	C5H4O	31.0184	**CH3O**
29.0027	**CHO**	81.034	**C5H5O**	32.0262	**CH4O**
30.0106	CH2O	85.029	**C4H5O2**	55.9898	C2O2
49.0289	*CH5O2*	87.0446	C4H7O2	58.0055	**C2H2O2**
55.0184	**C3H3O**	96.0211	C5H4O2	60.0211	**C2H4O2**
56.0262	**C3H4O**	*103.039*	*C4H7O3*	61.0289	**C2H5O2**
59.0133	**C2H3O2**	124.016	C6H4O3	72.0211	C3H4O2
59.0735	**C3H9N**	127.039	**C6H7O3**	73.029	**C3H5O2**
71.0133	C3H3O2	145.05	**C6H9O4**	74.0368	**C3H6O2**
				95.0133	C5H3O2

**BSOA**					
0.50 ≤ r < 0.70				0.70 ≤ r < 0.90	
m/z	*fragment ion*	*m/z*	*fragment ion*	*m/z*	*fragment ion*
39.0235	**C3H3**	106.042	C7H6O	43.0184	**C2H3O**
51.0235	C4H3	107.05	C7H7O	69.034	**C4H5O**
52.0313	C4H4	109.029	C6H5O2	81.034	**C5H5O**
53.0391	**C4H5**	109.065	C7H9O	82.0419	**C5H6O**
55.0184	**C3H3O**	113.024	C5H5O3	95.0497	C6H7O
58.0419	**C3H6O**	114.032	C5H6O3	96.0575	C6H8O
66.047	C5H6	122.037	C7H6O2	97.029	**C5H5O2**
67.0184	**C4H3O**	122.073	C8H10O	98.0368	C5H6O2
68.0262	C4H4O	123.081	C8H11O	99.0446	C5H7O2
70.0419	C4H6O	124.089	C8H12O	100.052	**C5H8O2**
71.0133	C3H3O2	125.024	C6H5O3	108.058	C7H8O
71.0497	**C4H7O**	127.039	**C6H7O3**	110.037	C6H6O2
74.0368	**C3H6O2**	127.076	**C7H11O2**	111.045	**C6H7O2**
78.047	C6H6	136.089	C9H12O	112.052	C6H8O2
79.0548	C6H7	140.084	C8H12O2	113.06	**C6H9O2**
82.0055	C4H2O2	141.092	C8H13O2	123.045	C7H7O2
83.0497	**C5H7O**	143.034	C6H7O4	124.052	**C7H8O2**
84.0575	C5H8O	148.089	C10H12O	125.06	C7H9O2
85.029	C4H5O2	150.068	C9H10O2	136.052	C8H8O2
86.0368	C4H6O2	155.034	C7H7O4	139.04	**C7H7O3**
87.0446	C4H7O2	157.05	C7H9O4	139.076	C8H11O2
94.0419	C6H6O	162.068	C10H10O2	141.055	**C7H9O3**
97.0653	**C6H9O**	167.071	C9H11O3	151.076	C9H11O2
101.024	C4H5O3	167.107	C10H15O2	153.055	C8H9O3
101.06	**C5H9O2**	169.087	C9H13O3	155.071	C8H11O3
				171.066	**C8H11O4**

**Haze** (0.50 ≤ r < 0.70)					
m/z	*fragment ion*	*m/z*	*fragment ion*	*m/z*	*fragment ion*
27.9949	CO	134.045	C4H8NO4	*148.016*	*C8H4O3*
104.026	C7H4O	146.037	C9H6O2		

OOA					
0.50 ≤ r < 0.70				0.70 ≤ r < 0.90	
m/z	*fragment ion*	*m/z*	*fragment ion*	*m/z*	*fragment ion*
31.0422	CH5N	101.024	C4H5O3	44.9977	CHO2
37.0078	C3H	102.032	C4H6O3	47.0133	CH3O2
38.0157	C3H2	113.024	C5H5O3	68.9977	C3HO2
40.0313	C3H4	123.008	C6H3O3	84.0211	C4H4O2
42.0106	C2H2O	125.024	C6H5O3	87.0082	C3H3O3
46.0055	CH2O2	137.024	C7H5O3	*87.0109*	*C6HN*
68.0262	C4H4O	139.003	C6H3O4	99.0082	C4H3O3
70.0055	C3H2O2	*140.011*	*C6H4O4*	100.016	C4H4O3
76.016	C2H4O3	*141.03*	*C5H5N2O3*	111.008	C5H3O3
86.9956	*C2HNO3*	*142.027*	*C6H6O4*	112.016	C5H4O3
88.016	C3H4O3	147.029	C5H7O5	115.018	C8H3O
89.0239	C3H5O3	153.019	C7H5O4	129.019	C5H5O4
100.004	*C3H2NO3*			*157.014*	*C6H5O5*

CHN-rich					
0.50 ≤ r < 0.70				0.70 ≤ r < 0.90	
m/z	*fragment ion*	*m/z*	*fragment ion*	*m/z*	*fragment ion*
29.0391	**C2H5**	94.0657	C6H8N	30.0344	**CH4N**
40.0187	C2H2N	95.0735	C6H9N	42.0344	**C2H4N**
41.0266	**C2H3N**	96.0813	C6H10N	43.0422	C2H5N
41.0391	C3H5	97.0891	C6H11N	44.05	**C2H6N**
43.0548	**C3H7**	98.061	C5H8NO	54.0344	C3H4N
45.0578	C2H7N	99.0684	C5H9NO	56.05	**C3H6N**
55.0422	C3H5N	99.1048	C6H13N	69.058	C4H7N
55.0548	**C4H7**	100.076	C5H10NO	70.0657	**C4H8N**
56.0626	C4H8	106.063	C4H10O3	82.066	C5H8N
57.0578	C3H7N	108.081	C7H10N	84.0813	C5H10N
57.0704	**C4H9**	110.097	C7H12N	86.061	C4H8NO
58.0657	**C3H8N**	112.064	C5H8N2O	98.097	C6H12N
68.05	C4H6N	112.113	C7H14N	*136.113*	*C9H14N*
71.0735	C4H9N	118.087	C5H12NO2	*138.116*	*C8H14N2*
72.0449	C3H6NO	122.097	C8H12N	*138.141*	*C10H18*
72.0813	**C4H10N**	124.113	C8H14N		
74.0606	C3H8NO	*126.092*	*C7H12NO*		
80.05	C5H6N	126.128	C8H16N		
81.058	C5H7N	*140.144*	*C9H18N*		
83.0735	C5H9N	*152.144*	*C10H18N*		
85.053	C4H7NO	*154.16*	*C10H20N*		
85.0891	*C5H11N*	175.112	C12H15O		
88.076	C4H10NO	*189.128*	*C13H17O*		

**POA** (0.50 ≤ r < 0.80)					
m/z	*fragment ion*	*m/z*	*fragment ion*	*m/z*	*fragment ion*
41.0391	C3H5	89.0391	**C7H5**	128.063	**C10H8**
42.047	**C3H6**	91.0548	**C7H7**	131.05	C9H7O
43.0548	C3H7	93.0704	**C7H9**	133.029	**C8H5O2**
55.0548	C4H7	95.0861	C7H11	135.117	C10H15
56.0626	C4H8	96.0939	C7H12	139.055	**C11H7**
67.0548	**C5H7**	97.1017	**C7H13**	144.057	C10H8O
68.0626	C5H8	98.0732	C6H10O	146.037	C9H6O2
69.0704	**C5H9**	101.039	**C8H5**	147.045	C9H7O2
70.0782	**C5H10**	104.026	**C7H4O**	*148.016*	*C8H4O3*
71.0861	**C5H11**	105.034	C7H5O	*150.047*	*C12H6*
73.0078	C6H	105.07	C8H9	151.055	**C12H7**
74.0156	**C6H2**	107.086	C8H11	152.063	**C12H8**
75.0235	**C6H3**	108.094	C8H12	160.052	C10H8O2
76.0313	**C6H4**	109.102	C8H13	*162.032*	*C9H6O3*
77.0391	**C6H5**	114.047	**C9H6**	165.07	**C13H9**
81.0704	C6H9	115.055	C9H7	*168.058*	*C12H8O*
82.0782	C6H10	118.042	C8H6O	176.063	**C14H8**
83.0861	**C6H11**	119.086	C9H11	180.058	**C13H8O**
84.0939	**C6H12**	121.102	**C9H13**	*181.059*	*C5H11NO6*
85.1017	**C6H13**	126.047	**C10H6**	185.097	C13H13O
87.0235	**C7H3**	127.055	**C10H7**	*189.055*	*C11H9O3*
88.0313	**C7H4**				

Table S6. Time series correlation between sulfate and haze or haze+OOA at the different stations during late winter/spring or for the entire datasets (block). Correlations are based on major ion measurements obtained in Université Grenoble Alpes, France. In cases where data from other institutes were utilized, this is indicated in parentheses behind the station acronym (ECCC: Environment and Climate Change Canada). ECCC data only from 2018 have been processed.

Station / Pearson's r	haze, block	haze, spring	haze+OOA, block	haze+OOA, spring	late winter/ spring months
ALT	0.771	0.814	0.823	0.871	Jan-May
ALT (ECCC)	0.812	0.957	0.805	0.937	Jan-May
BAR (Lomonosov Univ.)	0.655	0.436	0.734	0.834	Nov-Apr
GRU	0.742	0.796	0.829	0.931	Mar-May
PAL	0.528	0.788	0.683	0.861	Nov-May
TIK (Lomonosov Univ.)	*(low haze contrib.)*		0.775		
VRS	0.667	0.681	0.727	0.821	Mar-Jun
ZEP	0.446	0.700	0.807	0.867	Feb-Jun

Table S7. Time series correlation between AMS/PMF-based MSA-OA factor and available IC-based MSA data. Correlations are based on major ion measurements obtained in Université Grenoble Alpes, France. In cases where data from other institutes were utilized, this is indicated in parentheses behind the station acronym (ECCC: Environment and Climate Change Canada). ECCC data only from 2018 have been processed.

	MSA-OA (y) vs MSA (x)		
Station	Sample size	R^2	slope
ALT	47	0.84	0.86
ALT (ECCC)	25	0.93	0.86
BAR	6	0.84	1.23
UTQ (Baylor Univ.)	23	0.75	0.55
PAL	20	0.89	0.44
VRS	23	0.95	0.90
ZEP	22	0.95	0.55

Supplementary Figures

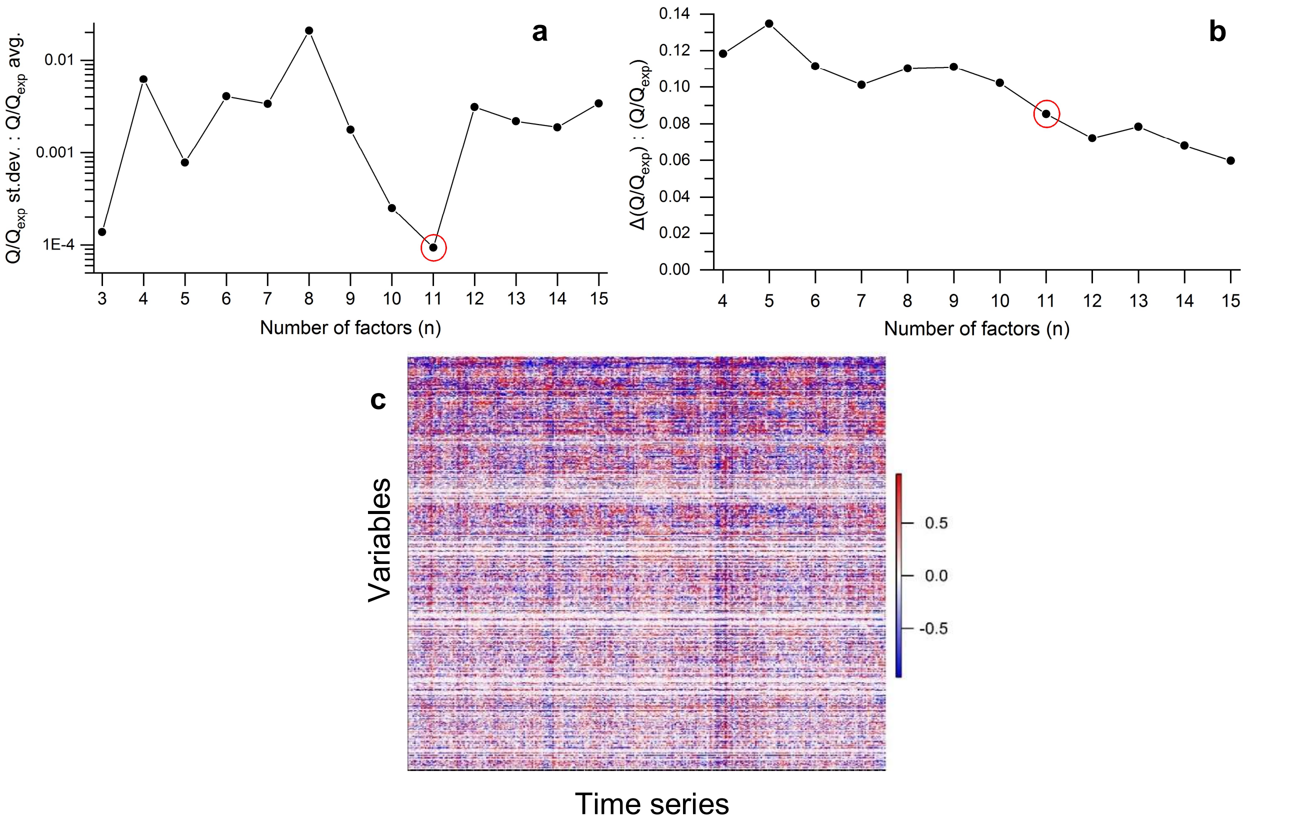

Figure S1. a) Diagnostic plot showing the relative Q/Q_{exp} standard deviation from five runs for *n*-factor solutions. b) Diagnostic plot showing the [$\Delta(Q/Q_{exp}$ avg.) / Q/Q_{exp} avg.] vs. the number of factors (*n*), from *n-1* to *n*-factor solutions, showing that solutions with more than 11 factors did not further reduce the residuals significantly (<10 %). c) Scaled residuals (Q/Q_{exp}; color scale on the right) of the base case 11-factor solution, for the full dataset (variables: HR fragment relative intensity).

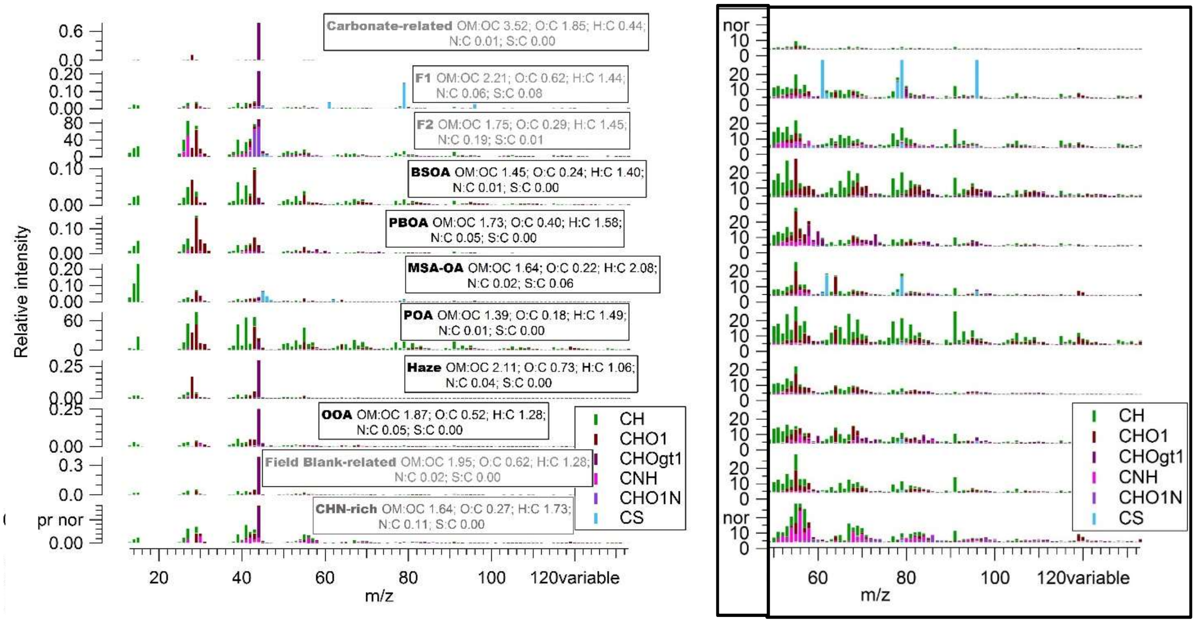

Figure S2. All 11-factor AMS/PMF mass spectra (profiles; shown as normalized fragment intensities) in HR with average atomic ratios, where the fragments are color-coded with the family. Factors with legends colored in grey (Carbonate-related, F1, F2, FB-related, CHN-rich) were interpreted to not be related to sampled organics or major OA sources. The spectra for *m/z* > 50 are magnified (right panel, x10^{-3}).

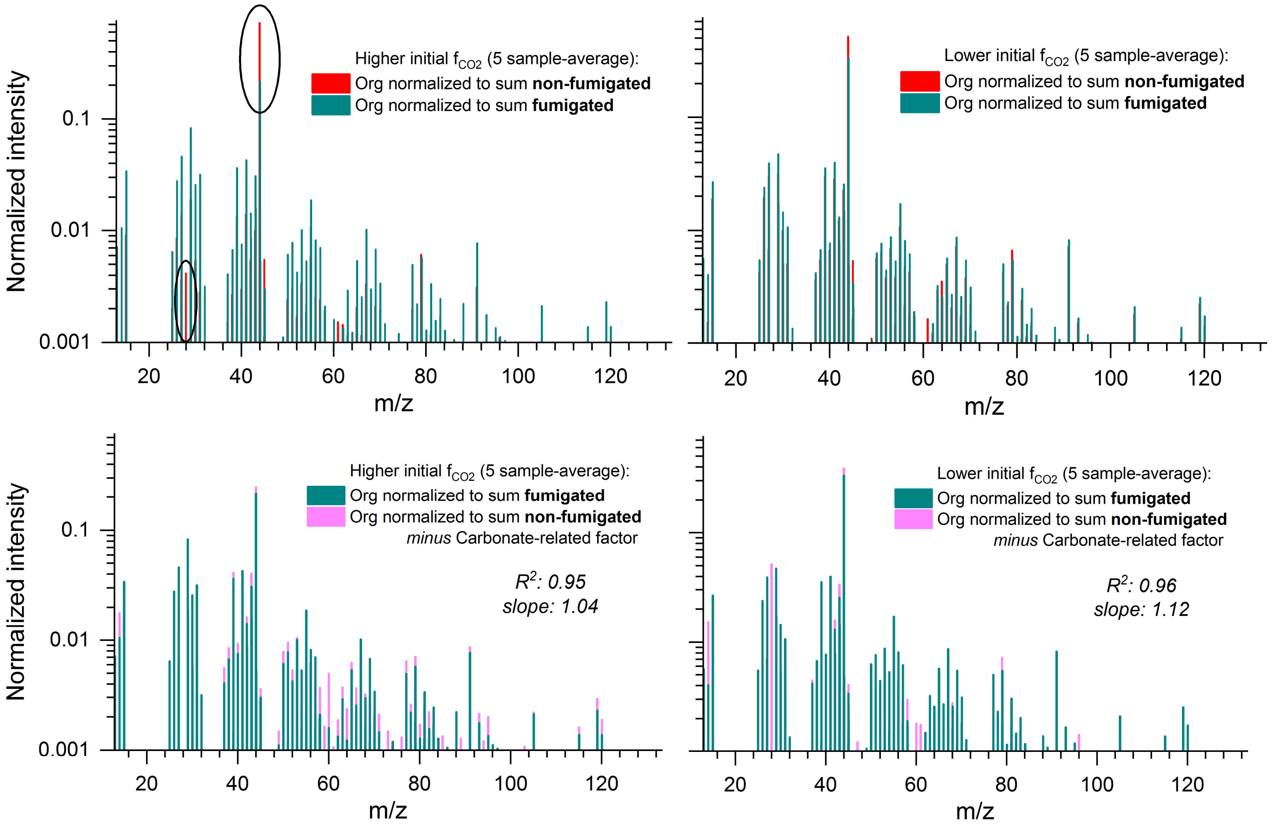

Figure S3. Comparison of normalized mass spectra, averaged for five samples with higher initial f_{CO2} and five other samples with lower initial f_{CO2}, with fumigation vs. without fumigation before the AMS measurement, as well as with fumigation vs. "without fumigation *minus* Carbonate-related factor". The former comparison indicates a substantial decrease in the CO/CO_2 signal upon fumigation in samples with larger initial f_{CO2}, while the latter comparison supports the mathematical subtraction of the Carbonate-related factor from the PMF analysis to account for the presence of inorganic carbonate in our samples.

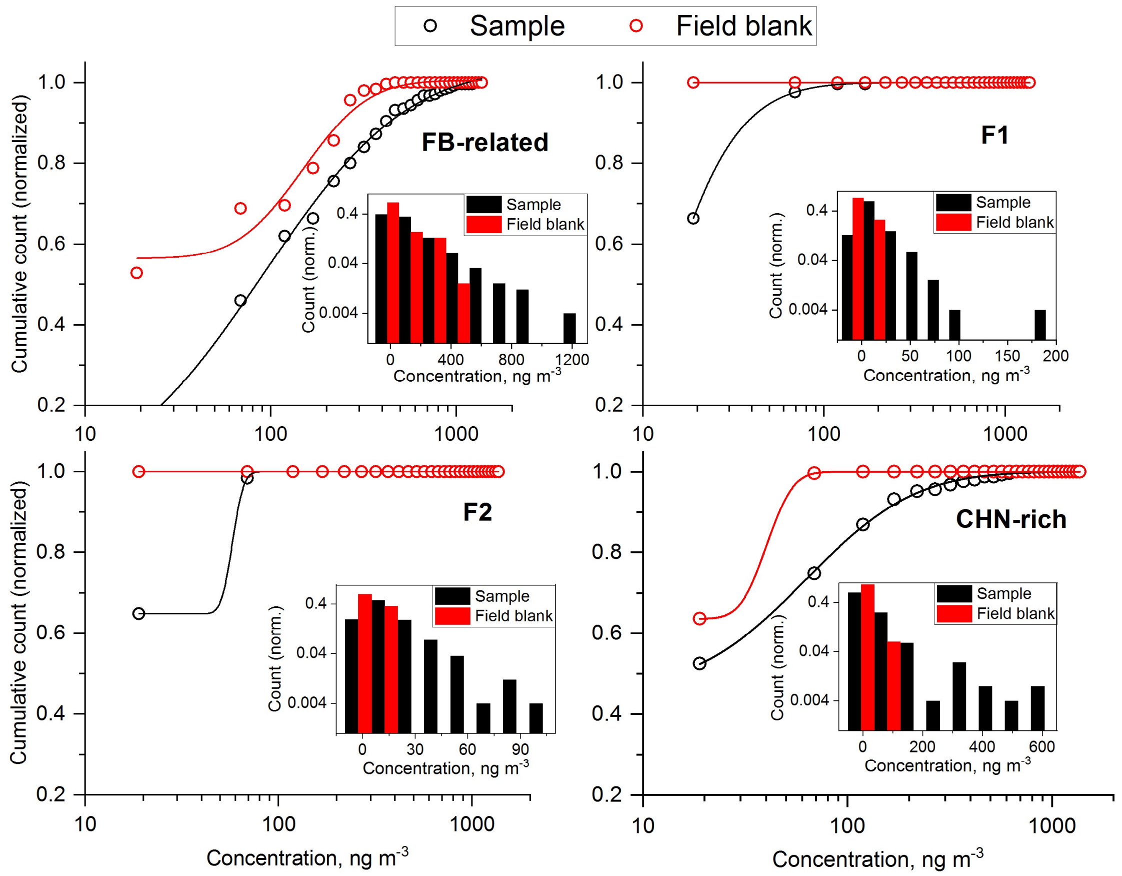

Figure S4. Normalized cumulative distribution functions (CDFs) for the water-extracted organic carbon mass concentration of the FB-related, F1, F2 and CHN-rich factors in the samples and in the blanks, for six stations (number of samples = 250) with available field blank filters. Open circles display actual data in thirty concentration bins (lines: fitted curves). Note that the same range of *x* and *y* variables is shown in all panels. Insets show normalized counts in log scale with different bin number for demonstration. The blank concentrations in each sample were estimated using station-specific (and season/year-specific, where applicable) blank-filter relative organic factor composition (from PMF) and the sample-specific m^3 of sampled air per cm^2 of filter area. Similar ranges and distributions were found for the FB-related factor. F1 and F2 did not exhibit concentrations higher than 100 ng m^{-3}. Together with CHN-rich, these factors did not have statistically different contributions in the samples from those in respective field blanks, i.e. [IQR] of day-to-day sample-to-blank mass ratio not statistically different from 1.0: FB-related, [0.7, 3]; F1, [1, 6]; F2, [1, 4], and CHN-rich, [0.9, 3].

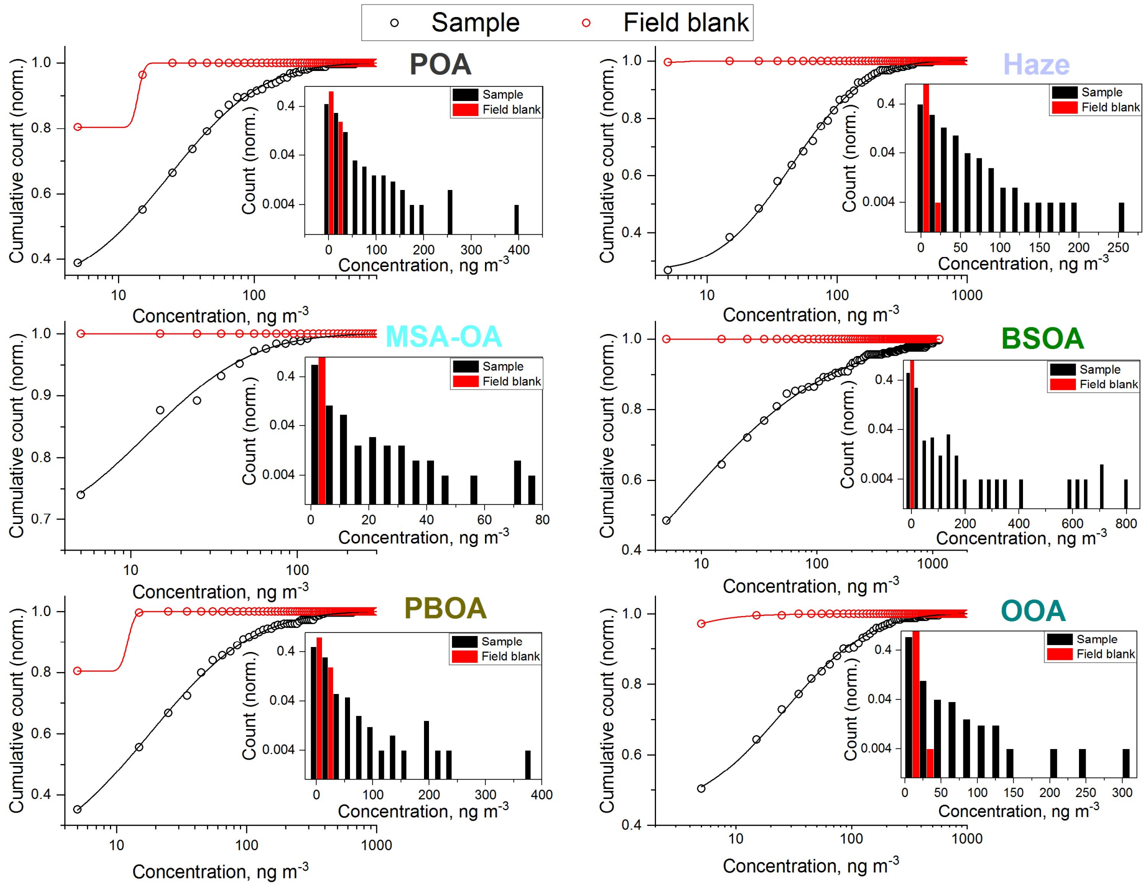

Figure S5. Normalized cumulative distribution functions (CDFs) for the mass concentrations of the six retained/interpretable WSOC factors in the samples and in the blanks, for six stations (number of samples = 250) with available field blank filters. Open circles display actual data with bin size 10 ng m^{-3} (lines: fitted curves). Insets show normalized counts in log scale with different bin size for demonstration. The blank concentrations in each sample were estimated based on station-specific (and season/year-specific, where applicable) blank-filter relative organic factor composition (from PMF) and the sample-specific m^3 of sampled air per cm^2. In contrast to the factors discussed in Fig. S4, these six factors did not contribute significantly to the signal of the field blanks. Specifically, all six factor concentrations in the blanks resided in the first or first two concentration bins (0-20 ng m^{-3}). Therefore, their detection limits were relatively low and their high concentrations in the samples can be considered real with high confidence. The full-dataset $P_{99,sample}$-to-$P_{99,blank}$ mass ratio for POA, haze, MSA-OA, BSOA, PBOA and OOA is 7, 19, 34, 93, 12 and 10, respectively (*P*: percentile).

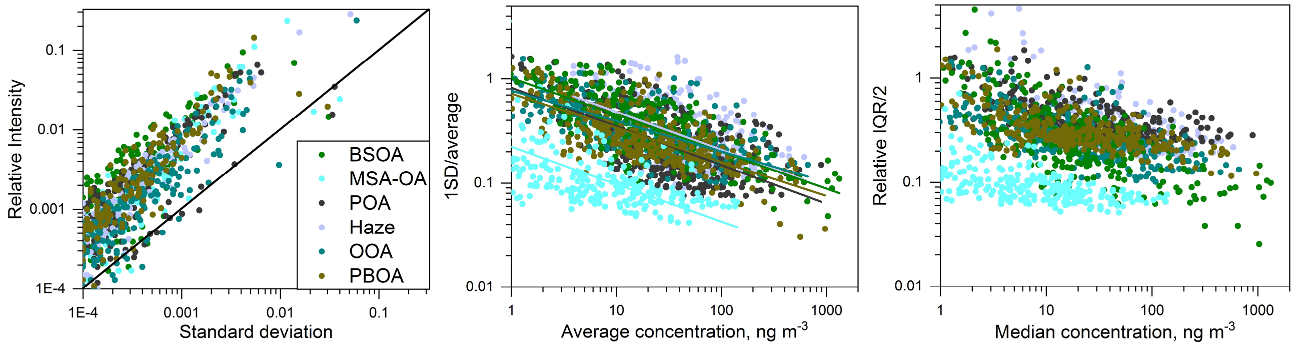

Figure S6. Scatter plot of the relative fragment intensities vs. their standard deviation (1 SD) for the six retained major WSOA factors from 100 BS runs, and the resulting time series of relative error (1SD/average or relative IQR/2) vs. the average or median factor concentrations. Black line shows the 1:1 line. Individual factor time series linear fits are shown with lines having the same color as the WSOA factor.

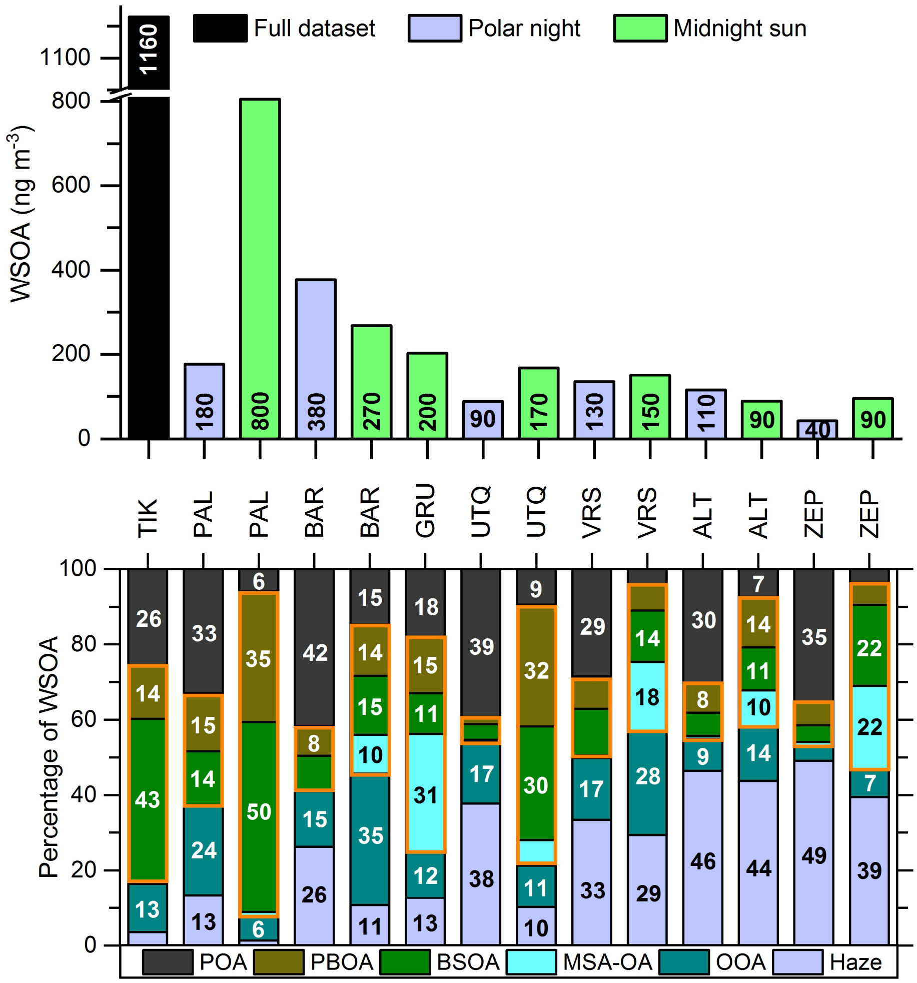

Figure S7. Station-specific seasonal absolute WSOA mass concentrations, sorted in descending order of the station annual-average OA, and the respective relative factor contributions to WSOA mass (before recovery corrections). The corresponding panels for total OA are found in the main text (Fig. 1).

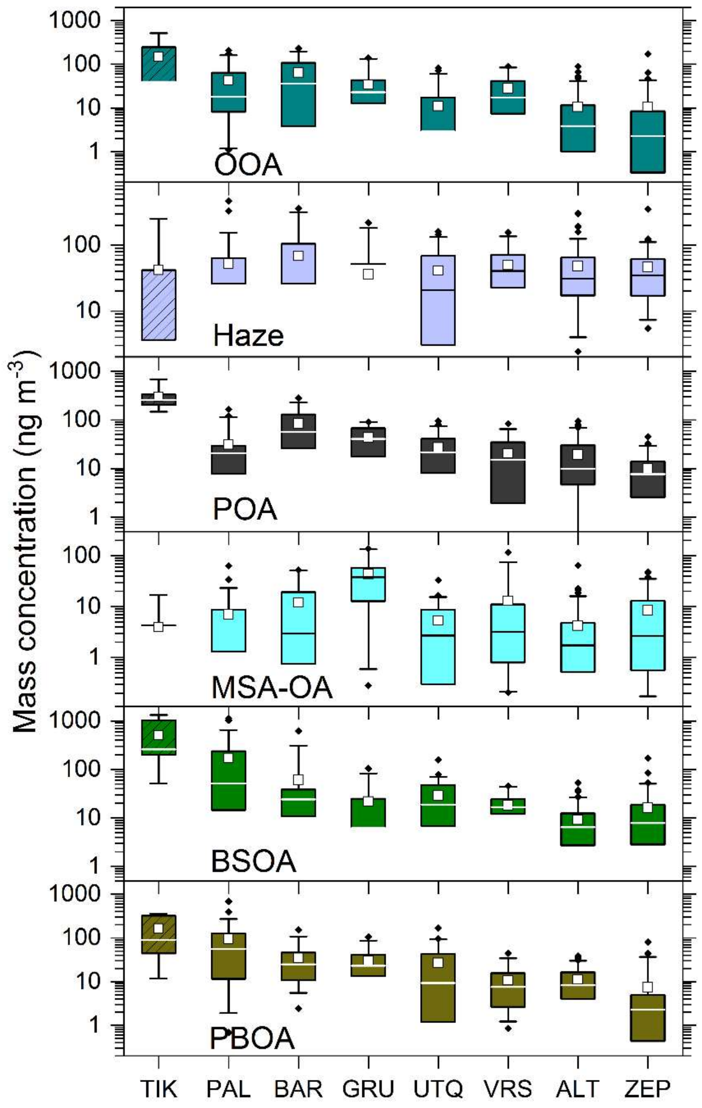

Figure S8. Same as Fig. 2, but for WSOA factors.

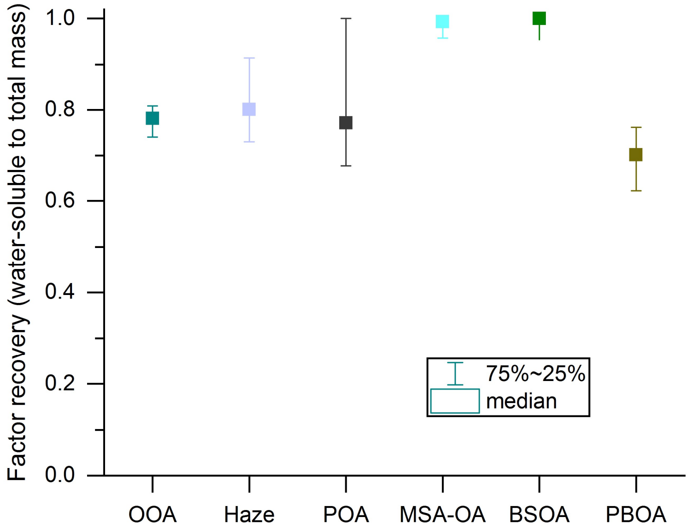

Figure S9. Estimated AMS/PMF recoveries for the major OA sources. The median values were used to convert the water-soluble to total OA mass.

Figure S10. Time series of cumulative (median) absolute factor contributions to total OA mass at each station (median composite dates shown for the sampling period at each station from start to end).

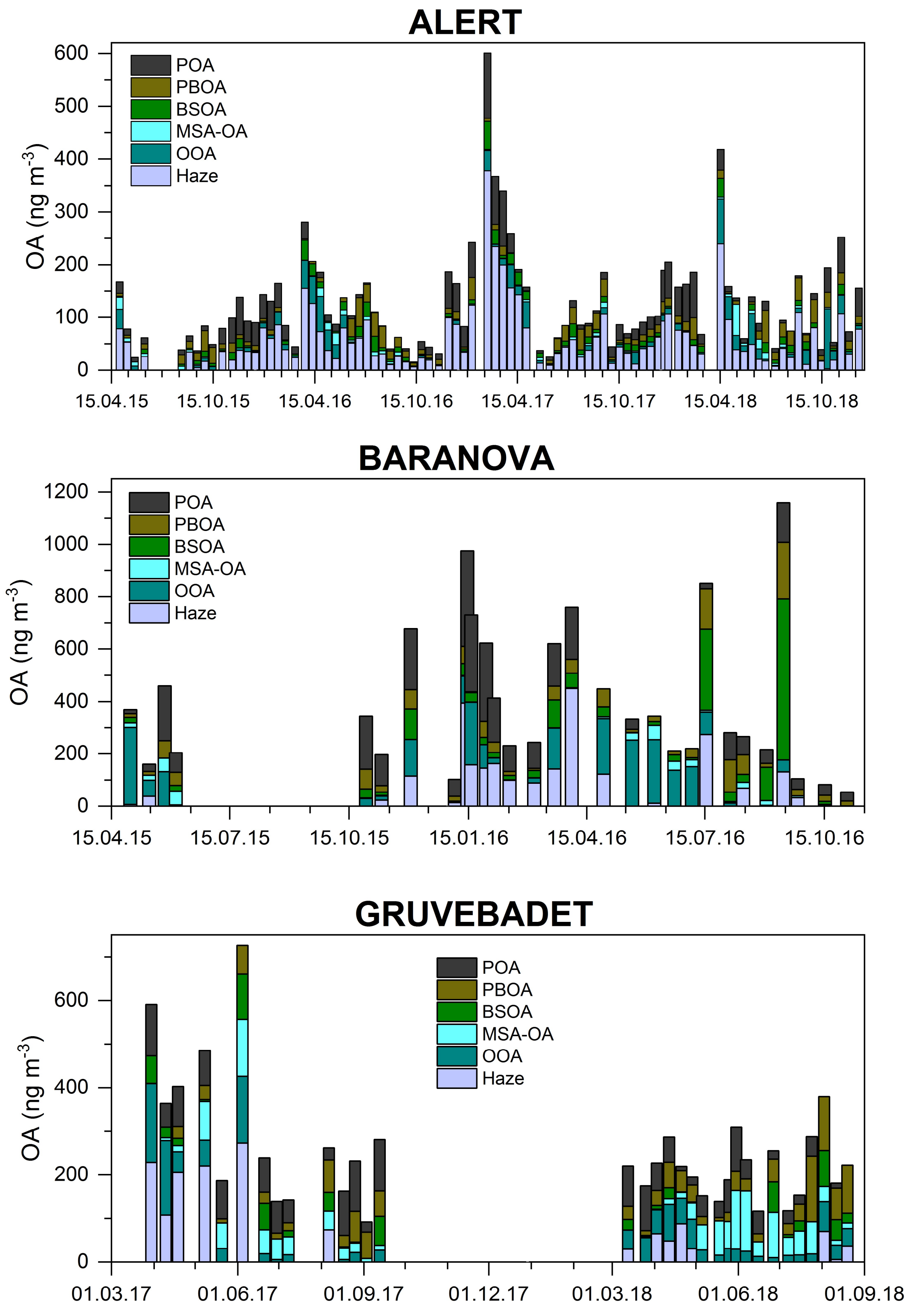

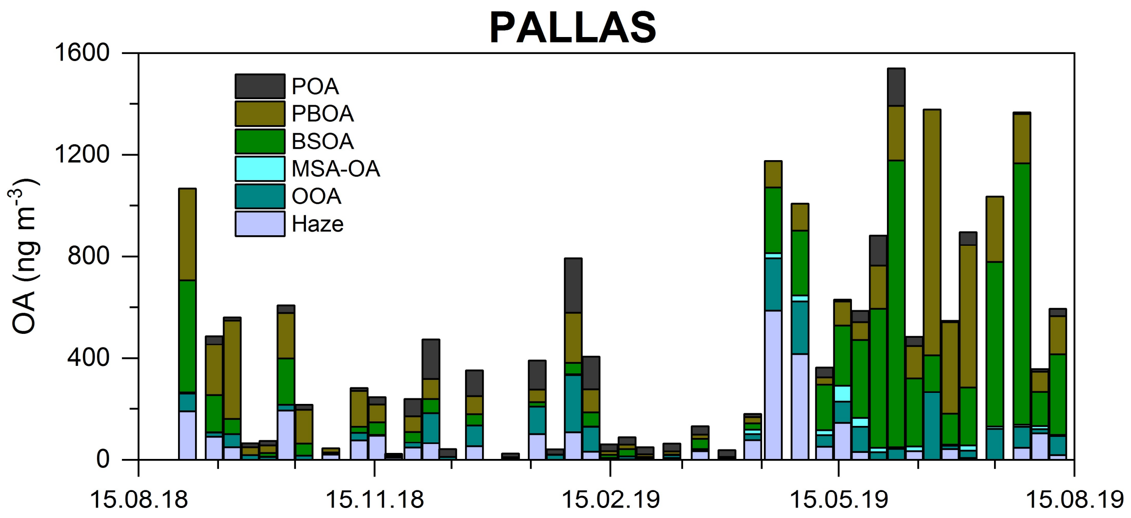
PALLAS
POA
PBOA
BSOA
MSA-OA
OOA
Haze
OA (ng m^{-3})
0
400
800
1200
1600
15.08.18
15.11.18
15.02.19
15.05.19
15.08.19

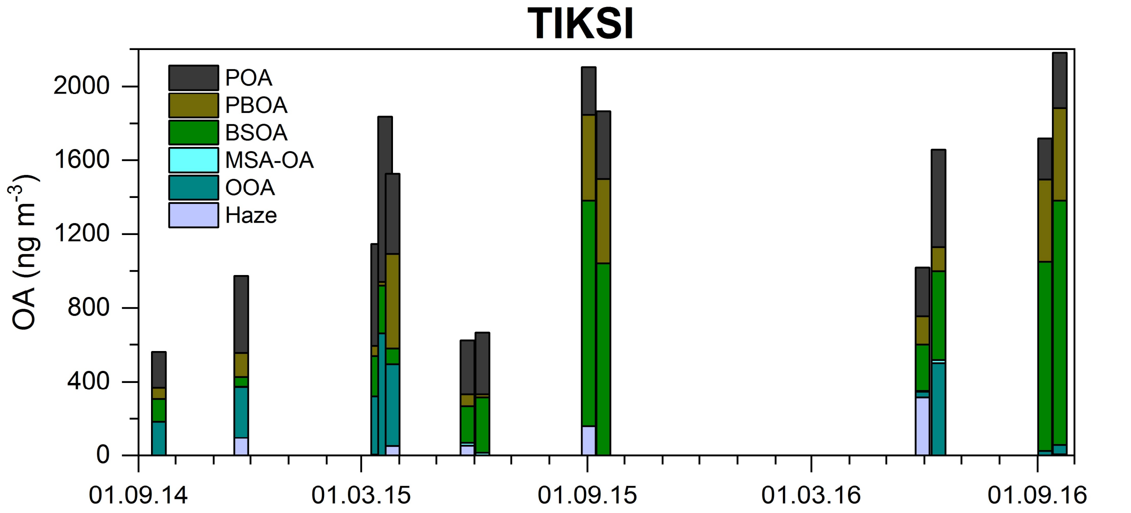
TIKSI
POA
PBOA
BSOA
MSA-OA
OOA
Haze
OA (ng m^{-3})
0
400
800
1200
1600
2000
01.09.14
01.03.15
01.09.15
01.03.16
01.09.16

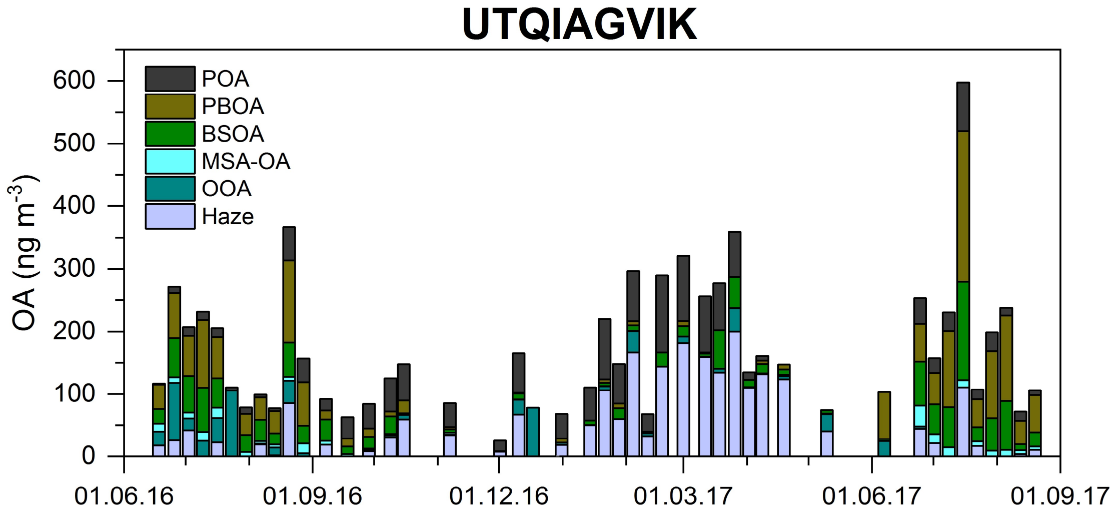
UTQIAGVIK
POA
PBOA
BSOA
MSA-OA
OOA
Haze
OA (ng m^{-3})
0
100
200
300
400
500
600
01.06.16
01.09.16
01.12.16
01.03.17
01.06.17
01.09.17

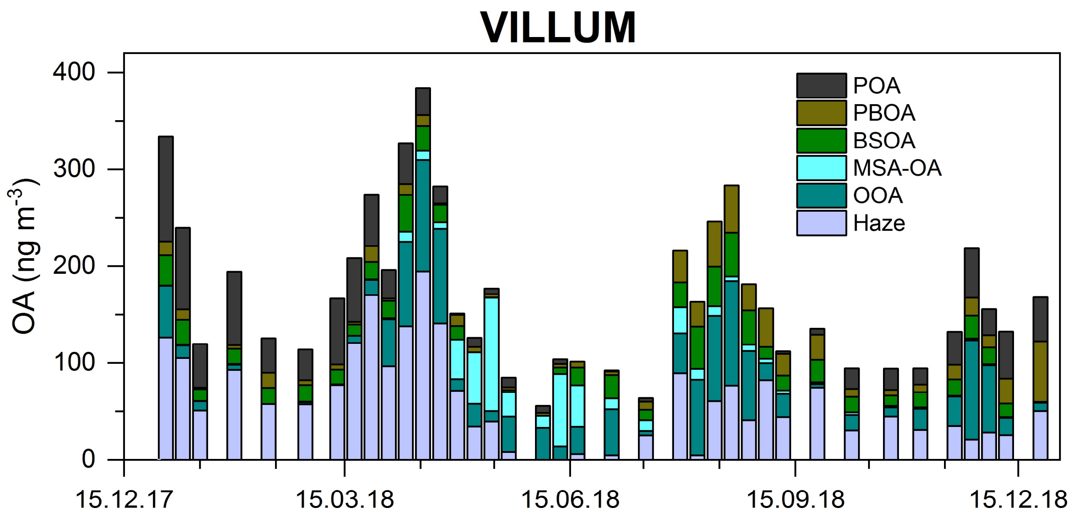
VILLUM
OA (ng m-3)
400
300
200
100
0
15.12.17
15.03.18
15.06.18
15.09.18
15.12.18
POA
PBOA
BSOA
MSA-OA
OOA
Haze

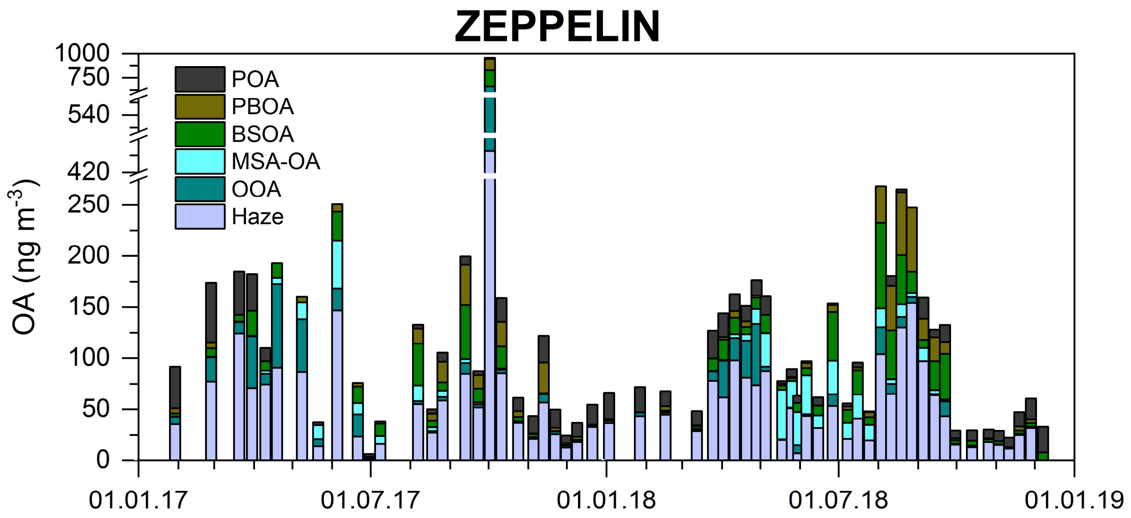
ZEPPELIN
OA (ng m-3)
1000
750
540
420
250
200
150
100
50
0
01.01.17
01.07.17
01.01.18
01.07.18
01.01.19
POA
PBOA
BSOA
MSA-OA
OOA
Haze

Figure S11. Back-trajectory analysis results using ZeFir, based on concentration-weighted trajectories (CWT), where the entire time series of each WSOA factor mass from each station were used as input. The receptor site is indicated with a red circle. The heat maps indicate air parcels responsible for high measured factor concentrations arriving at a receptor site (label), and thus potential major source regions for the associated long-term datasets. Results for stations with very low year-long factor concentrations that are not omitted here should be interpreted with caution.

i) Haze:

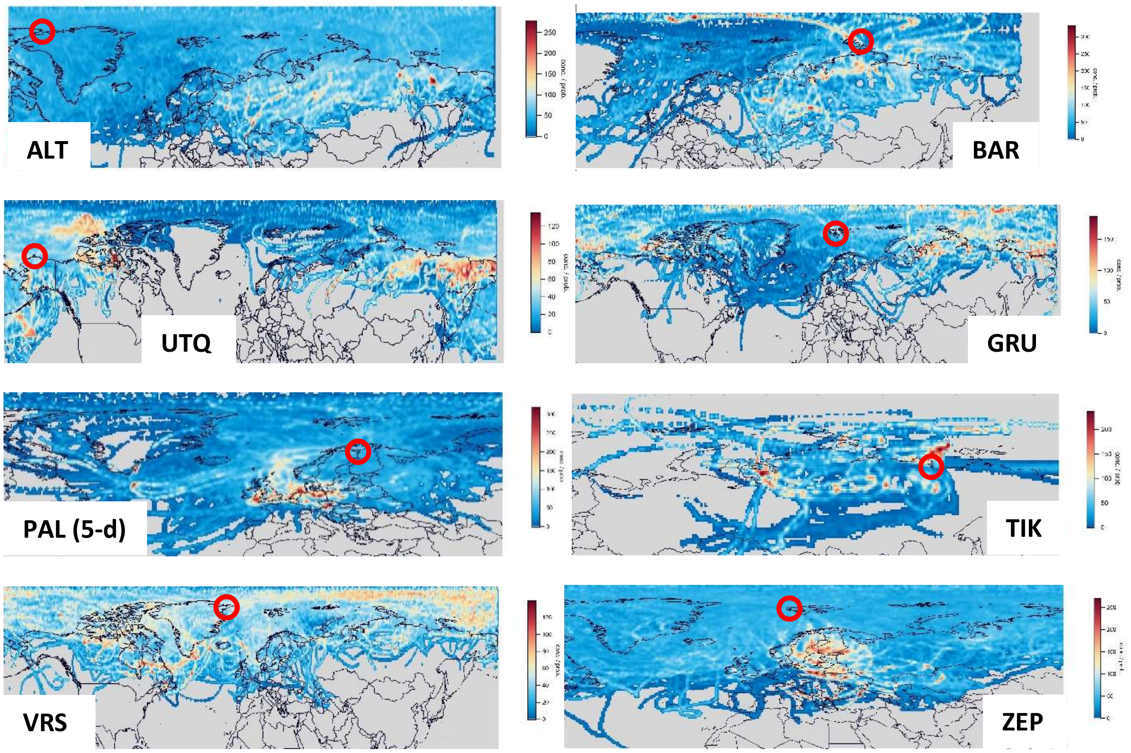

ii) POA:

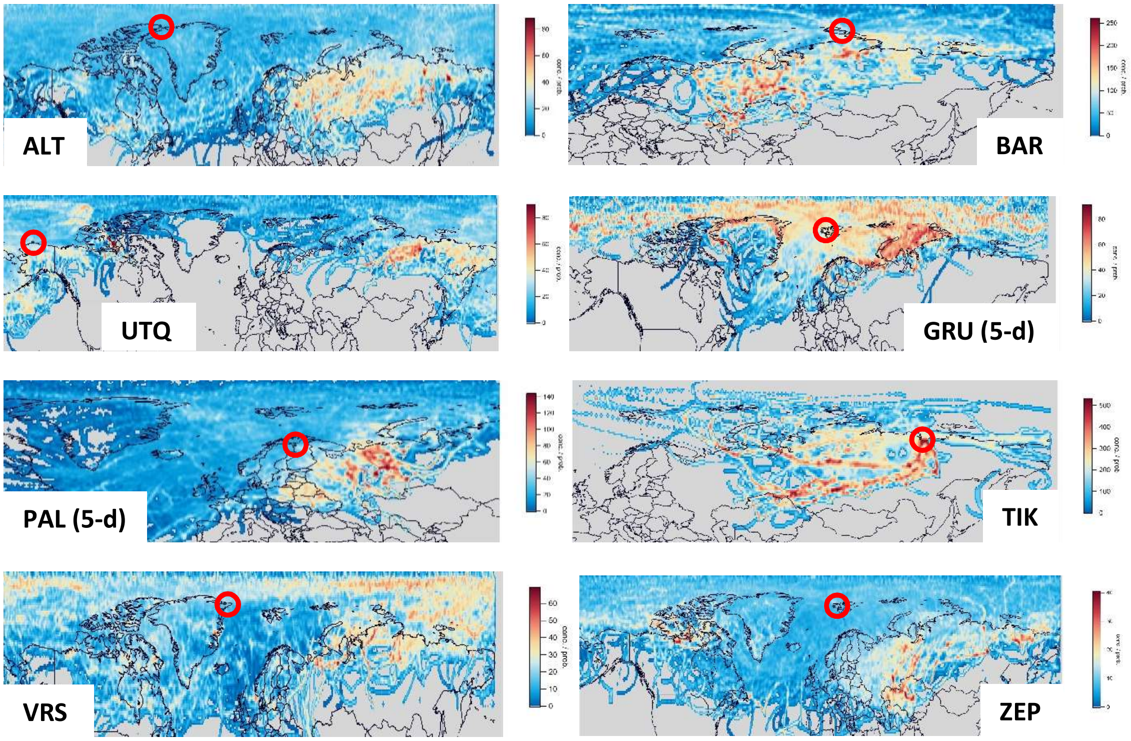

iii) MSA-OA:

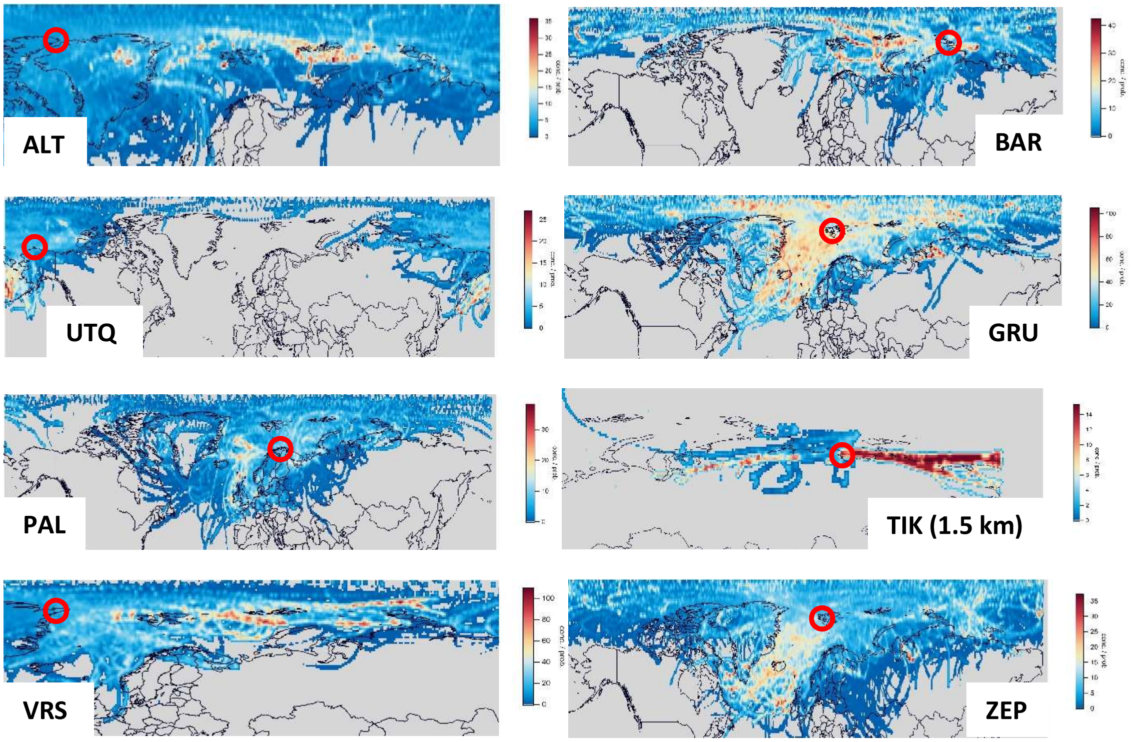

iv) BSOA:

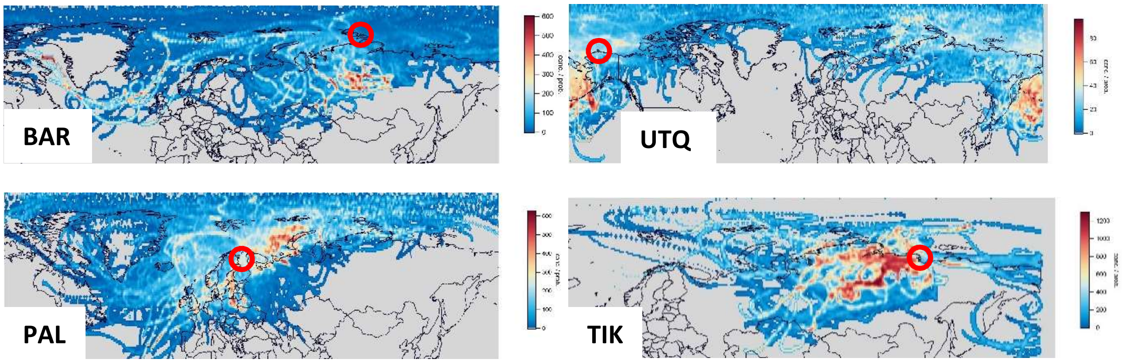

PBOA (all sites merged, 5-d BTs, 3 km; ALT/ZEP: 1.5 km):

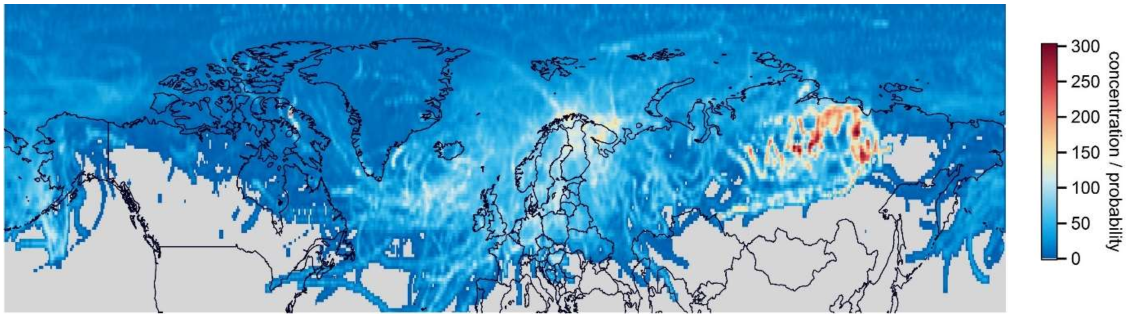

OOA (merged sites except for TIK, 10-d BTs, 3 km):

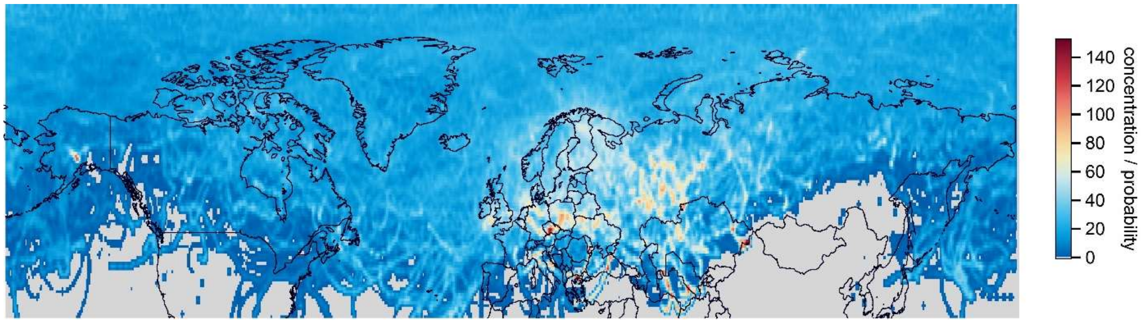

OOA (TIK, 10-d BTs, 3 km):

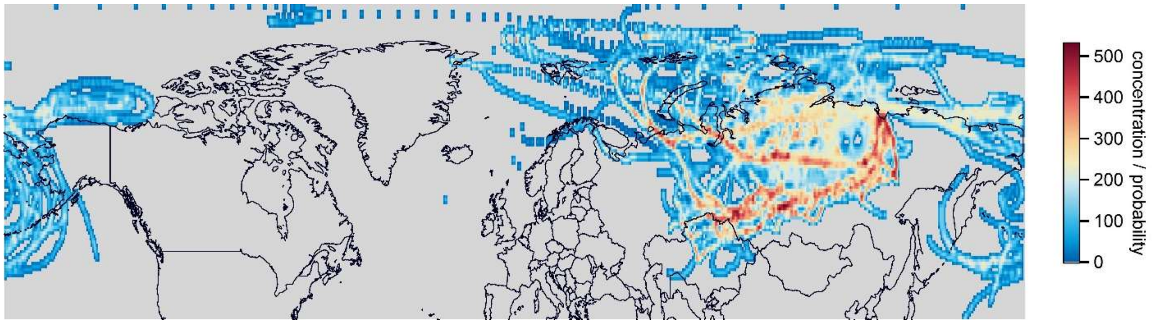

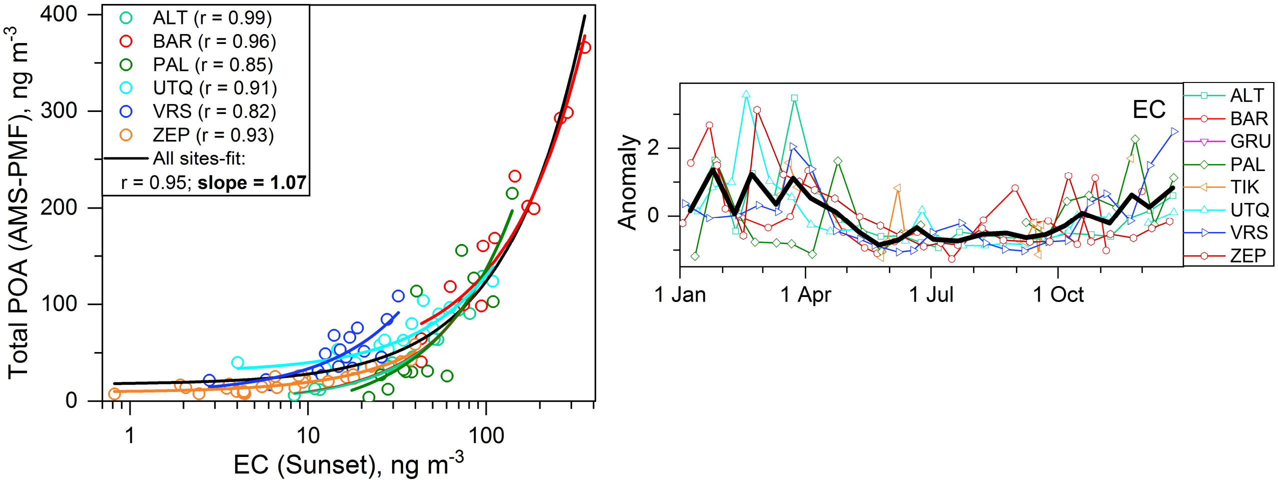

Figure S12. Left panel: correlation between total POA factor concentration and Sunset-EC at six stations with available winter samples, when maximum concentration was observed for both species, e.g. ALT 17 (a few outliers were not considered for the linear fits: 3.5% of 115 winter samples). The strong linear correlation might imply a similar source for these species, assuming that the Arctic EC is predominantly of fossil origin in winter and that the fossil fraction of EC remains relatively constant during this period. Specifically, using the POA OM:OC (Fig. S2) and assuming that around 75 % of wintertime EC originates from oil/gas extraction, our estimated source-specific POC:EC mass ratio of ~ 1.1 is remarkably similar to that estimated by Peters et al. (2011) for the same OC/EC source emission in the Arctic during 2004[50]. Right panel: seasonal cycle of EC at all stations, which can be compared to that of POA in Fig. 3.

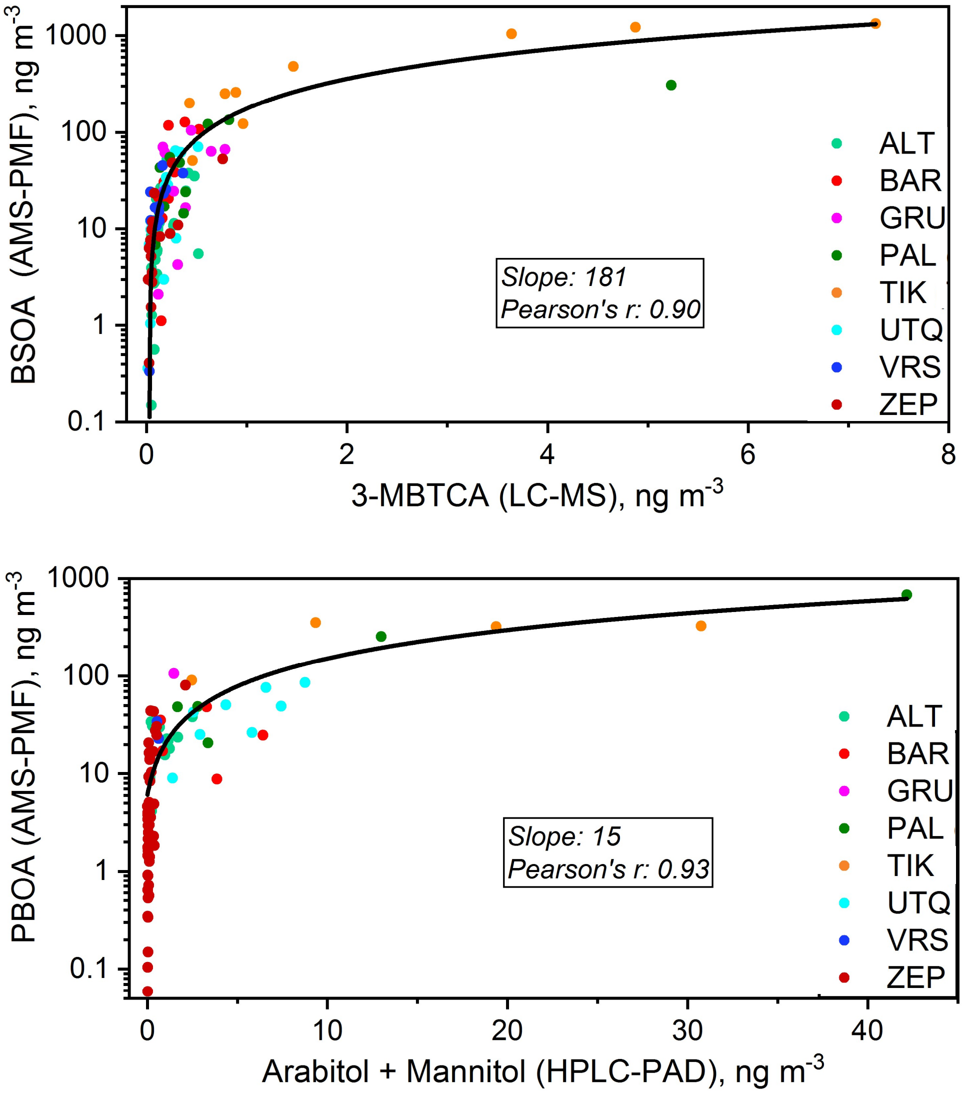

Figure S13. Linear correlations between water-soluble AMS/PMF factor (*y*-axes) and external organic marker (*x*-axes) concentrations. The latter were measured in Université Grenoble Alpes (France) for a subset of samples used for AMS/PMF.

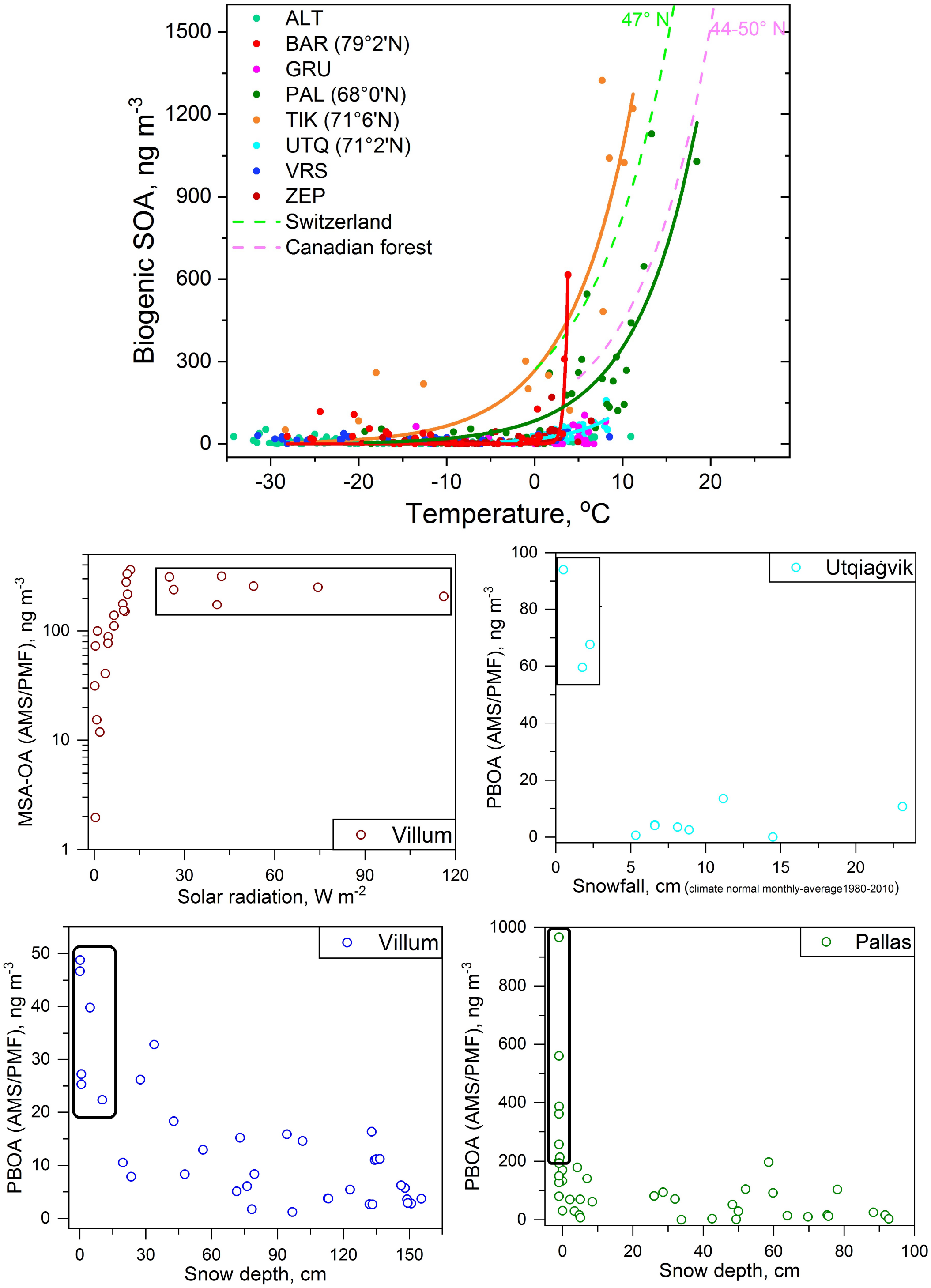

Figure S14. Effect of environmental parameters [temperature (*T*), solar radiation, snow] on the different natural organic fractions (BSOA, MSA-OA, PBOA). Note the different range of the *y*-axis concentrations. Exponential fits are based on the equation $y = y_0 * \exp(A * x)$. The fitted parameter *A* (R^2) for Switzerland[17] (PM_{10} Magadino and Zurich 2013; daily-average T range: 0-30 °C), the Canadian forest[51], PAL, TIK and UTQ is 0.112 (0.66), 0.125 (0.62), 0.187 (0.76), 0.140 (0.75) and 0.198 (0.50), respectively. Temperature from Kenttärova (forest site ~1 km from PAL-Matorova) is shown for PAL from mid-June to mid-August (8 samples; correlation with PAL-Matorova *T* for the other data points, slope: 1.00, R^2: 0.99). Offsets from the Canadian/PAL fitted curves and significant presence of BSOA at lower (than expected) temperatures (e.g. high-latitude BAR), might indicate more regional influence (see also back-ward trajectory analysis for BSOA at BAR/TIK). Snow depth data from Kenttärova are shown for PAL (no available measurements from Matorova). Significant presence of PBOA mass at high snow depths might indicate more regional influence, resuspension or measurement errors for low factor concentrations. Note that the effective albedo in Svalbard typically drops from 0.8 (snow-covered surface) in March to 0.1 (uncovered tundra ground) by July[52], when we observe the onset of PBOA at GRU and ZEP (Fig. 3).

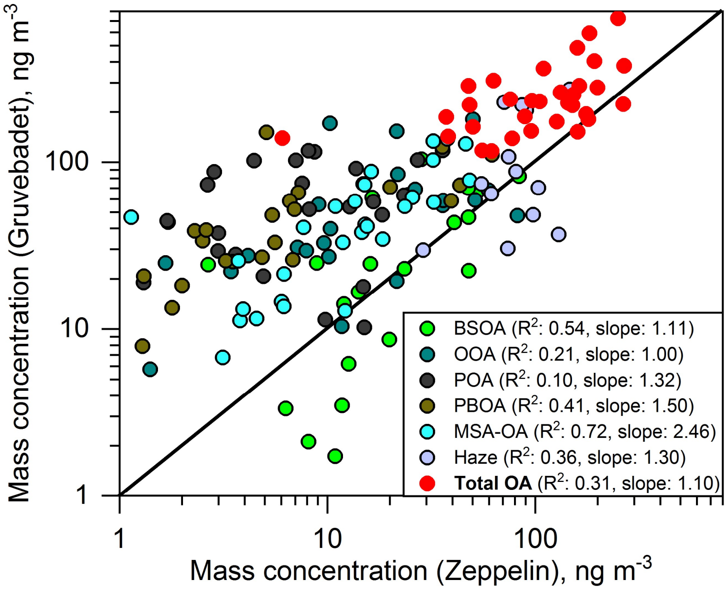

Figure S15. Heterogeneity in OA (internal) composition in the Arctic: correlation between OA factor time series and total OA (almost concurrent data; max. 1d difference between the two sites) in high-altitude ZEP vs. sea-level GRU stations (both in Svalbard). The thick black line corresponds to 1:1 relationship. Absence of correlations indicates different OA composition in both absolute and relative terms. Similarity in seasonal trends is observed only for MSA-OA, in line with similar source regions indicated by back-ward trajectory analysis (Fig. S11).

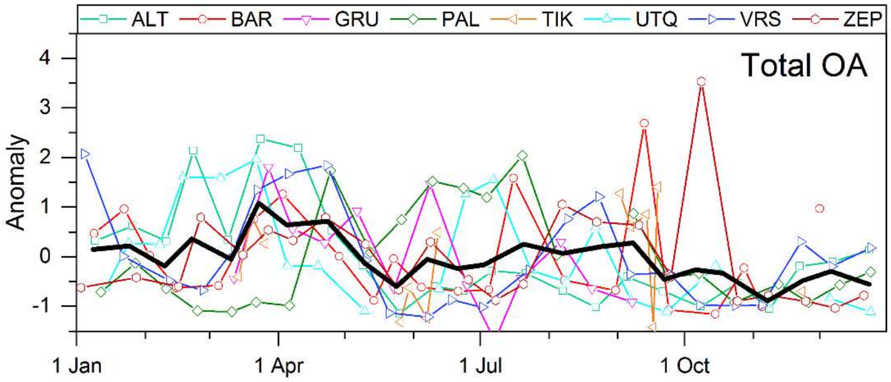

Figure S16. Same as Fig. 3, but for total OA (sum of 6 factors).

Supplementary References

1. Manousakas M., *et al.* Aerosol carbonaceous, elemental and ionic composition variability and origin at the Siberian High Arctic, Cape Baranova. *Tellus B Chem Phys Meteorol.* **72**, 1-14 (2020).
2. Caiazzo L., *et al.* Carbonaceous aerosol in polar areas: first results and improvements of the sampling strategies. *Atmosphere.* **12**, (2021).
3. Lohila, 2015; Preface to the special issue on integrated research of atmosphere, ecosystems and environment at Pallas; *Boreal Environment Research* 20: 431–454.
4. Cavalli et al., 2010; www.atmos-meas-tech.net/3/79/2010.
5. Pieber S. M., *et al.* Inorganic salt interference on $CO_{2(+)}$ in Aerodyne AMS and ACSM organic aerosol composition studies. *Environ. Sci. Technol.* **50**, 10494-10503 (2016).
6. Jaffrezo et al., 2005; www.atmos-chem-phys.org/acp/5/2809.
7. Jaffrezo et al., 1998; Atmospheric Environment Vol. 32, No. 14/15, pp. 2705Ð2708, 1998.
8. Waked A., *et al.* Source apportionment of PM_{10} in a north-western Europe regional urban background site (Lens, France) using positive matrix factorization and including primary biogenic emissions. *Atmos. Chem. Phys.* **14**, 3325-3346 (2014).

9. Chan T. W., *et al.* Inter-comparison of elemental and organic carbon mass measurements from three North American national long-term monitoring networks at a co-located site. *Atmos. Meas. Techn.* **12**, 4543-4560 (2019).
10. Huang L., *et al.* Stable isotope measurements of carbon fractions (OC/EC) in airborne particulate: A new dimension for source characterization and apportionment. *Atmos. Environ.* **40**, 2690-2705 (2006).
11. Huang, L., Zhang, W., Santos, G. M., Rodríguez, B. T., Holden, S. R., Vetro, V., and Czimczik, C. I.: Application of the ECT9 protocol for radiocarbon-based source apportionment of carbonaceous aerosols, *Atmos. Meas. Tech.* **14**, 3481–3500 (2021).
12. Popovicheva O., *et al.* East Siberian Arctic background and black carbon polluted aerosols at HMO Tiksi. *Sci. Total Environ.* **655**, 924-938 (2019).
13. Vlachou A., *et al.* Development of a versatile source apportionment analysis based on positive matrix factorization: a case study of the seasonal variation of organic aerosol sources in Estonia. *Atmos. Chem. Phys.* **19**, 7279-7295 (2019).
14. Hall and Horowitz, 1996; Econometrica, 1996, vol. 64, issue 4, 891-916.
15. Politis D. N. & White H. Automatic Block-Length Selection for the Dependent Bootstrap. *Econometric Reviews.* **23**, 53-70 (2004).
16. Norris and Henry, 2007; EPA Unmix 6.0 Fundamentals & User Guide; EPA/600/R-07/089 June 2007 www.epa.gov.
17. Daellenbach K. R., *et al.* Long-term chemical analysis and organic aerosol source apportionment at nine sites in central Europe: source identification and uncertainty assessment. *Atmos. Chem. Phys.* **17**, 13265-13282 (2017).
18. Hedberg E., Gidhagen L. & Johansson C. Source contributions to PM_{10} and arsenic concentrations in Central Chile using positive matrix factorization. *Atmos. Environ.* **39**, 549-561 (2005).
19. Jankowski N., Schmidl C., Marr I. L., Bauer H. & Puxbaum H. Comparison of methods for the quantification of carbonate carbon in atmospheric PM_{10} aerosol samples. *Atmos. Environ.* **42**, 8055-8064 (2008).
20. Canagaratna M. R., *et al.* Chemical and microphysical characterization of ambient aerosols with the Aerodyne aerosol mass spectrometer. *Mass Spectrom. Rev.* **26**, 185-222 (2007).
21. Bozzetti C., *et al.* Organic aerosol source apportionment by offline-AMS over a full year in Marseille. *Atmos. Chem. Phys.* **17**, 8247-8268 (2017).
22. Scalabrin E., *et al.* Amino acids in Arctic aerosols. *Atmos. Chem. Phys.* **12**, 10453-10463 (2012).
23. Mashayekhy Rad F., *et al.* Measurements of atmospheric proteinaceous aerosol in the Arctic using a selective UHPLC/ESI-MS/MS strategy. *J. Am. Soc. Mass Spectrom.* **30**, 161-173 (2019).
24. Feltracco M., *et al.* Free and combined L- and D-amino acids in Arctic aerosol. *Chemosphere.* **220**, 412-421 (2019).
25. Schneider J., *et al.* Mass-spectrometric identification of primary biological particle markers and application to pristine submicron aerosol measurements in Amazonia. *Atmos. Chem. Phys.* **11**, 11415-11429 (2011).
26. Nicolescu T. O. Interpretation of Mass Spectra. *Mass Spectrometry*, 2017.
27. Zorn et al., 2008; www.atmos-chem-phys.net/8/4711/2008.
28. Schmale J., *et al.* Sub-Antarctic marine aerosol: dominant contributions from biogenic sources. *Atmos. Chem. Phys.* **13**, 8669-8694 (2013).
29. Huang D. D., Li Y. J., Lee B. P. & Chan C. K. Analysis of organic sulfur compounds in atmospheric aerosols at the HKUST supersite in Hong Kong using HR-ToF-AMS. *Environ. Sci. Technol.* **49**, 3672-3679 (2015).
30. Mallet M. D., *et al.* Summertime surface PM_1 aerosol composition and size by source region at the Lampedusa island in the central Mediterranean Sea. *Atmos. Chem. Phys.* **19**, 11123-11142 (2019).
31. Bozzetti C., *et al.* Size-resolved identification, characterization, and quantification of primary biological organic aerosol at a European rural site. *Environ. Sci. Technol.* **50**, 3425-3434 (2016).
32. Faiola C. L., Wen M. & VanReken T. M. Chemical characterization of biogenic secondary organic aerosol generated from plant emissions under baseline and stressed conditions: inter- and intra-species variability for six coniferous species. *Atmos. Chem. Phys.* **15**, 3629-3646 (2015).
33. Pauraite J., *et al.* Characterization of aerosol mass spectra responses to temperature over a forest site in Lithuania. *J. Aerosol Sci.* **133**, 56-65 (2019).
34. Robinson N. H., *et al.* Evidence for a significant proportion of Secondary organic aerosol from isoprene above a maritime tropical forest. *Atmos. Chem. Phys.* **11**, 1039-1050 (2011).
35. Xu L., Guo H., Boyd C. M., Klein M., Bougiatioti A, *et al.* Effects of anthropogenic emissions on aerosol formation from isoprene and monoterpenes in the southeastern United States. *Proc. Natl. Acad. Sci. USA.* **112**, 37-42 (2015).
36. Kuwata M., Liu Y., McKinney K. & Martin S. T. Physical state and acidity of inorganic sulfate can regulate the production of secondary organic material from isoprene photooxidation products. *Phys. Chem. Chem. Phys.* **17**, 5670-5678 (2015).
37. Riva M., *et al.* Chemical characterization of secondary organic aerosol from oxidation of isoprene hydroxyhydroperoxides. *Environ. Sci. Technol.* **50**, 9889-9899 (2016).
38. Brüggemann M., *et al.* Real-time detection of highly oxidized organosulfates and BSOA marker compounds during the F-BEACh 2014 field study. *Atmos. Chem. Phys.* **17**, 1453-1469 (2017).
39. Kostenidou E., Karnezi E., Kolodziejczyk A., Szmigielski R. & Pandis S. N. Physical and chemical properties of 3-methyl-1,2,3-butanetricarboxylic Acid (MBTCA) aerosol. *Environ. Sci. Technol.* **52**, 1150-1155 (2018).
40. Mass spectral fragments of common hydrocarbons; http://www.hidenanalytical.com/wp-content/uploads/2016/08/hydrocarbon_fragments-1-1.pdf.

41. Silverstein, R, G. Bassler and T. Morrill. Spectrometric identification of organic compounds. New York, NY. John Wiley and Sons. Inc.
42. Stohl A. Characteristics of atmospheric transport into the Arctic troposphere. *J. Geophys. Res. Atmos.* **111**, (2006).
43. Kahl J. D. W.*, et al.* Air mass trajectories to Summit, Greenland: a 44-year climatology and some episodic events. *J. Geophys. Res. Oceans.* **102**, 26861-26875 (1997).
44. Buch et al., 2019: Arctic *in situ* data availability.
45. Rozwadowska et al., 2010; www.atmos-chem-phys.net/10/877/2010.
46. Parker M. D., Dahl J. M. L. & Wicker L. J. Uncertainties in trajectory calculations within near-surface mesocyclones of simulated supercells. *Mon. Weather Rev.* **140**, 2959-2966 (2012).
47. Esau and Sorokina, 2010: Climatology of the Arctic planetary boundary layer.
48. Kahl, 1993: Atmospheric Environment Vol. 27A, No, 17/18, pp. 3037-3038, 1993.
49. Cheng M.-D. Geolocating Russian sources for Arctic black carbon. *Atmos. Environ.* **92**, 398-410 (2014).
50. Peters G. P.*, et al.* Future emissions from shipping and petroleum activities in the Arctic. *Atmos. Chem. Phys.* **11**, 5305-5320 (2011).
51. Leaitch W. R.*, et al.* Temperature response of the submicron organic aerosol from temperate forests. *Atmos. Environ.* **45**, 6696-6704 (2011).
52. Maturilli M., Herber A. & König-Langlo G. Surface radiation climatology for Ny-Ålesund, Svalbard (78.9° N), basic observations for trend detection. *Theor. Appl. Climatol.* **120**, 331-339 (2014).
